# Oleanolic Acid Dimers with Potential Application in Medicine—Design, Synthesis, Physico-Chemical Characteristics, Cytotoxic and Antioxidant Activity

**DOI:** 10.3390/ijms25136989

**Published:** 2024-06-26

**Authors:** Andrzej Günther, Przemysław Zalewski, Szymon Sip, Piotr Ruszkowski, Barbara Bednarczyk-Cwynar

**Affiliations:** 1 Department of Organic Chemistry, Faculty of Pharmacy, Poznan University of Medical Sciences, Collegium Pharmaceuticum 2 (CP.2), Rokietnicka Str. 3, 60-806 Poznan, Poland; bcwynar@ump.edu.pl; 2 Department of Pharmacognosy and Biomaterials, Faculty of Pharmacy, Poznan University of Medical Sciences, Collegium Pharmaceuticum 1 (CP.1), Rokietnicka Str. 3, 60-806 Poznan, Poland; pzalewski@ump.edu.pl (P.Z.); szymonsip@ump.edu.pl (S.S.); 3 Department of Pharmacology and Phytochemistry, Institute of Natural Fibres and Medicinal Plants, Wojska Polskiego Str. 71b, 60-630 Poznan, Poland; 4 Department of Pharmacology, Faculty of Pharmacy, Poznan University of Medical Sciences, Collegium Pharmaceuticum 1 (CP.1), Rokietnicka Str. 3, 60-806 Poznan, Poland; pruszkowski@gmail.com; 5 Center of Innovative Pharmaceutical Technology (CITF), Rokietnicka Str. 3, 60-806 Poznan, Poland

**Keywords:** triterpenes, oleanolic acid, triterpene dimers, oleanolic acid dimers, SAR, cytotoxic activity, antioxidant activity

## Abstract

The present work aimed to obtain a set of oleanolic acid derivatives with a high level of cytotoxic and antioxidant activities and a low level of toxicity by applying an economical method. Oleanolic acid was alkylated with α,ω-dihalogenoalkane/α,ω-dihalogenoalkene to obtain 14 derivatives of dimer structure. All of the newly obtained compounds were subjected to QSAR computational analysis to evaluate the probability of the occurrence of different types of pharmacological activities depending on the structure of the analysed compound. All dimers were tested for cytotoxicity activity and antioxidant potential. The cytotoxicity was tested on the SKBR-3, SKOV-3, PC-3, and U-87 cancer cell lines with the application of the MTT assay. The HDF cell line was applied to evaluate the tested compounds’ Selectivity Index. The antioxidant test was performed with a DPPH assay. Almost all triterpene dimers showed a high level of cytotoxic activity towards selected cancer cell lines, with an IC_50_ value below 10 µM. The synthesised derivatives of oleanolic acid exhibited varying degrees of antioxidant activity, surpassing that of the natural compound in several instances. Employing the DPPH assay, compounds **2a**, **2b**, and **2f** emerged as promising candidates, demonstrating significantly higher Trolox equivalents and highlighting their potential for pharmaceutical and nutraceutical applications. Joining two oleanolic acid residues through their C-17 carboxyl group using α,ω-dihalogenoalkanes/α,ω-dihalogenoalkenes resulted in the synthesis of highly potent cytotoxic agents with favourable SIs and high levels of antioxidant activity.

## 1. Introduction

Chemical compounds of natural origin are becoming increasingly popular among scientists [1]. This interest concerns the possibility of isolating chemical compounds from plant material, their chemical modifications, and the pharmacological activity of both groups of substances (i.e., natural and modified). Obtaining new derivatives of natural compounds through chemical modifications is one of the most compelling trends that interests scientists considerably. The aim of carrying out chemical modifications is to obtain compounds with a higher level of pharmacological activity than their parent substances or obtain compounds that will demonstrate new directions of pharmacological activity and other utilitarian properties.

**Figure 1:**
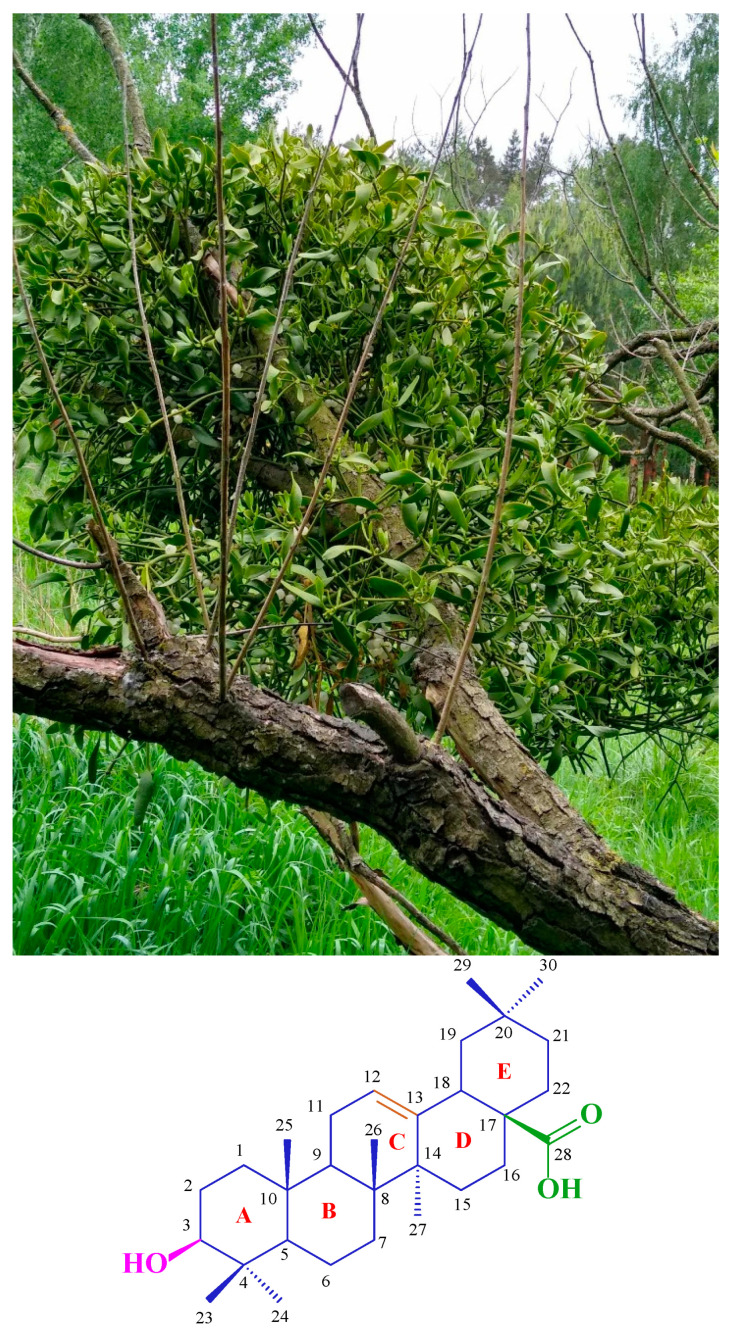
One of the sources of oleanolic acid (**1**) and the structure of this compound.

Among natural compounds, flavonoids, alkaloids, and terpenoids are very popular [2]. The last of these, terpenoids, also known as isoprenoids, are substances widely distributed in the plant world. They constitute an extensive group of substances—over 40,000 [3]—divided into several subgroups depending on the molecule’s structure. The largest and most popular group among terpenoids is triterpenes, characterised by skeletons always composed of 30 carbon atoms. Oleanolic acid (Figure 1) represents triterpenes with an oleanane skeleton, occurring in at least 1600 edible and medicinal plants [4]. A rich source of this compound is, among others, mistletoe herb (*Viscum alba*, Figure 1). Its carbon skeleton consists of five six-membered rings, as shown in Figure 1. There is a hydroxyl group at the C-3 position of the oleanane skeleton, a carboxyl group at the C-17 position, and a double bond between the C-12 and C-13 atoms. The presence of the three mentioned reactive functional groups in the oleanolic acid molecule means that the compound can be subjected to numerous chemical modifications, producing numerous derivatives.

The growing interest in triterpenes, including oleanolic acid, has resulted in the appearance of a vast number of publications in the scientific literature demonstrating various directions of the pharmacological activity of this acid in recent years. Numerous scientific studies have shown, among others, anticomplementary [5], antileishmanial [6], antimycobacterial [7], antioxidative [8], antitumour and anti-HIV [9], antiatherosclerotic [10], antioxidant and anti-inflammatory [11], antihypertensive [12], antihypolipidemic [13], anticancer [14], and other properties. Notably, great hopes are associated with the cytotoxic activity of oleanolic acid and its numerous derivatives [14]. The anticancer activity of oleanolic acid has been demonstrated against four human liver cancer cell lines HepG2, Hep3B, HUH7, and HA22T [15], the human colon carcinoma cell line HCT15 [16], ovarian carcinoma IGROV1 and breast cancer cell line MDA-MB-231 [17], lung cancer cell lines [18], hepatocellular carcinoma cell line HuH7 [19], and many others.

The primary research trend in our department is chemical modifications of oleanolic acid with expected cytotoxic activity against various cancer cell lines. Experimental work published so far demonstrates a high level of cytostatic activity, primarily against KB, MCF-7, HeLa, Hep-G2, and 549 cancer cell lines [20,21,22,23,24].

Oxidative stress plays a significant role in the pathogenesis of numerous diseases such as liver disorders, inflammation, tumours, and diabetes, in which OA treatment has been discovered to be beneficial [25]. Reactive oxygen species (ROS, e.g., superoxide ^•^O_2_^−^, hydroxyl ^•^OH^−^ and others), also known as free radicals, are continuously produced by the body’s regular use of oxygen, such as respiration and some cell-mediated immune functions [26]. ROS at physiological concentrations are necessary for normal cell function. However, suppose cellular constituents do not effectively scavenge ROS. In that case, excess ROS can react with many biomolecules such as DNA [27], lipids [28], and proteins [29], initiating the peroxidation of membrane lipids, leading to the accumulation of lipid peroxides and the damage of DNA and proteins, and finally resulting in disease conditions. Oleanolic acid probably generates its protective effects mainly through antioxidant mechanisms, quenching ROS, inhibiting lipid peroxidation, or indirectly stimulating cellular antioxidant defences [30].

Several triterpenoid compounds and their derivatives have been shown to demonstrate promising antioxidant properties in experimental and clinical studies, mostly from the ursane, oleanane, and lupane groups (e.g., [31,32,33,34]).

In this work, we present the results of the latest experimental work on the synthesis of a new type of oleanolic acid derivatives named OADs (Oleanolic Acid Dimers), their cytostatic activity against SKBR-3 (human breast adenocarcinoma), SKOV-3 (human ovarian cystadenocarcinoma), PC-3 (human prostate carcinoma), and U-87 (human glioblastoma) cell lines, and their antioxidant activity.

## 2. Results

### 2.1. Synthesis of OADs

**Figure 2:**
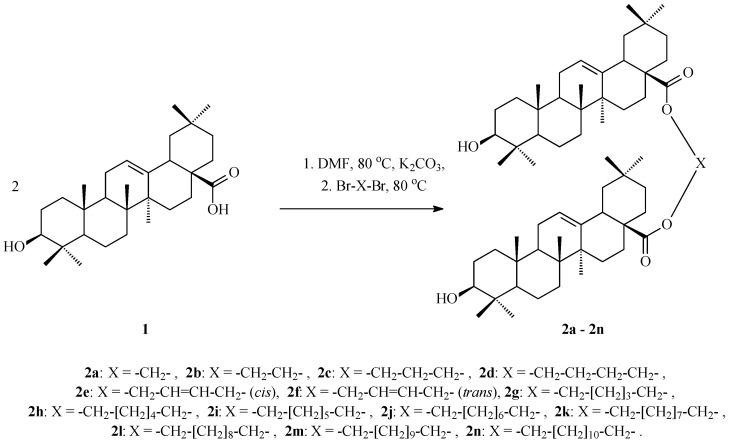
Synthesis of OADs **2a**–**2n**.

Chemical transformations of oleanolic acid (**1**) leading to its dimers (Oleanolic Acid Dimers, OADs) **2a**–**2n** are shown in Figure 2.

### 2.2. Structure–Activity Relationship Analysis

The highest results of the structure–activity relationship (SAR) analysis for oleanolic acid (**1**) and OADs **2a**–**2n** are given in Table 1 and Table 2. The detailed results of the SAR analysis with P_a_ ≥ 0.700 are given in Supplementary File S1 (Table S1).

### 2.3. Cytotoxic Activity of the Obtained OADs ***2a***–***2n***

#### 2.3.1. In Vitro Assay

The cytotoxic activity of oleanolic acid (mother compound, **1**, Figure 1) and 14 synthesised OADs (**2a**–**2n**, Figure 2) was tested against four carcinoma cell lines, SKBR-3 (human breast adenocarcinoma), SKOV-3 (human ovarian cystadenocarcinoma), PC-3 (human prostate carcinoma), and U-87 (human glioblastoma), and against one normal cell line, HDF (regular fibroblast cell line). This activity was evaluated in vitro using the standard MTT method (enzymatic reduction of 3-[4,5-dimethylthiazole-2-yl]-2,5-diphenyltetrazolium bromide, MTT, to MTT-formazan) based on a viability assay [21]. The estimated half-maximal inhibitory concentrations (IC_50_) for the above-mentioned triterpenes (**1** and **2a**–**2n**) are presented in Table 3.

#### 2.3.2. Selectivity Index

The results of the Selectivity Index (SI), calculated as IC_50_ for the regular cell line (HDF)/IC_50_ for the respective cancerous cell line, are given in Table 4.

### 2.4. Antioxidant Activity

**Figure 3:**
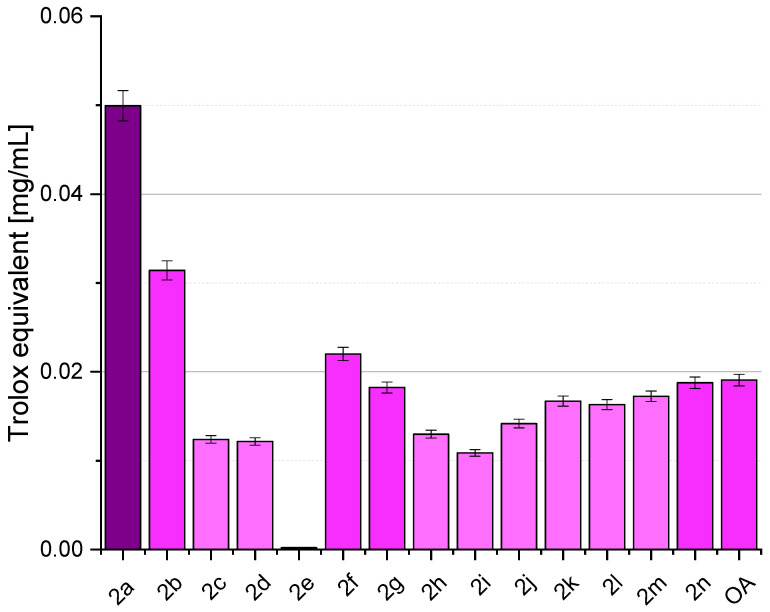
The ability of OADs **2a**–**2n** and oleanolic acid (**1**, OA) to inhibit the DPPH radical, expressed as Trolox equivalent.

**Figure 4:**
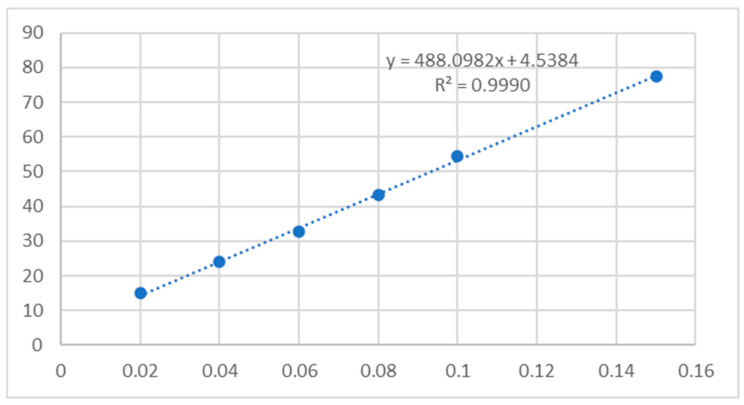
Standard curve for DPPH radical inhibition by Trolox.

Figure 3 depicts various samples’ DPPH radical-scavenging activity, with OA (oleanolic acid, **1**) representing the naturally occurring compound and subsequent entries representing synthesised OADs **2a**–**2n**. The results are presented as % inhibition of the DPPH radical and Trolox equivalent, calculated from the standard curve (Figure 4). Activity assays were performed in eight repetitions.

## 3. Discussion

### 3.1. Synthesis

#### 3.1.1. Synthesis of Cytotoxic Agents **2a**–**2n**

The cytotoxic agents **2a**–**2n** were obtained from oleanolic acid (**1**) using the carboxyl group at the C-17 position of this acid.

Since dimers of oleanolic acid obtained through the C-17 carboxyl group and a linker with an odd number of carbon atoms are unknown, we first decided to test the possibility of obtaining such a derivative containing only one carbon atom in the linker. The resulting derivative of this type would have a free hydroxyl group at the C-3 position, enabling the synthesis of numerous further derivatives.

Oleanolic acid (**1**) was dissolved in dried dimethylformamide (DMF) by stirring the flask’s contents and heating it to 80 °C. A two-fold excess of potassium carbonate was added, and stirring and heating were continued. Then, methylene iodide was added dropwise, and stirring and heating were continued for a further 30 min.

As a result of the developed method of oleanolic acid (**1**) dimerisation, crude product **2a** was obtained with over 90% yield. This is much higher than the yield from the literature concerning the synthesis of oleanolic acid with a two-carbon linker [35]. The significantly higher yield of dimer **2a** compared to the cited literature is associated with the use of better selected reaction parameters, primarily the triterpene/dihalogenoalkane/-alkene molar ratio, which is a key element determining not only the high yield of the reaction but also the high degree of purity of the obtained dimer. The appropriate choice of the above proportion is also associated with other advantages of the developed method, namely a significant reduction in cost and labour consumption.

Since TLC control of the crude dried product showed no impurities or by-products, column purification of the dimer was unnecessary. The whitish product was purified by crystallisation from ethanol with water. White crystalline needles melted at 183–185 °C.

The synthesis of the Oleanolic Acid Dimer with a two-carbon linker (**2b**) was carried out similarly to the one-carbon linker (**2a**).

The synthesis of the remaining dimers with both short and long linkers (**2c**–**2n**) was carried out according to the above method. The reaction time from the dropwise addition of the α,ω-dihalogenoalkane/α,ω-dihalogenoalkene was always 30 min. Only one product was obtained in each reaction, so the post-reaction mixture did not need to be purified on a silica gel chromatography column. The crude product was crystallised from ethanol or, if crystallisation failed, precipitated with water from an ethanolic solution. In this way, 14 dimers of oleanolic acid (Oleanolic Acid Dimers, OADs, **2a**–**2n**) were obtained, of which 2 dimers contained a four-carbon linker, one double bond between the second and third carbon atoms of the linker, one in the *cis* position (dimer **2e**), and the other in the *trans* position (**2f**).

#### 3.1.2. The Dependency between Linker Length and Melting Point

All OADs (**2a**–**2n**, Figure 2) were crystallised from ethanol (in the case of dimer **2a** from ethanol with water) after being isolated from the mixture after the reaction. Ethyl alcohol is one of the best solvents used for the crystallisation of oleanolic acid derivatives, although there are some cases where crystallisation attempts using this solvent failed. To make it easier to compare the susceptibility of OADs to crystallisation, we decided to carry out this process only with ethanol and, in the case of failure, to precipitate the product with water from an ethanol solution.

**Figure 5:**
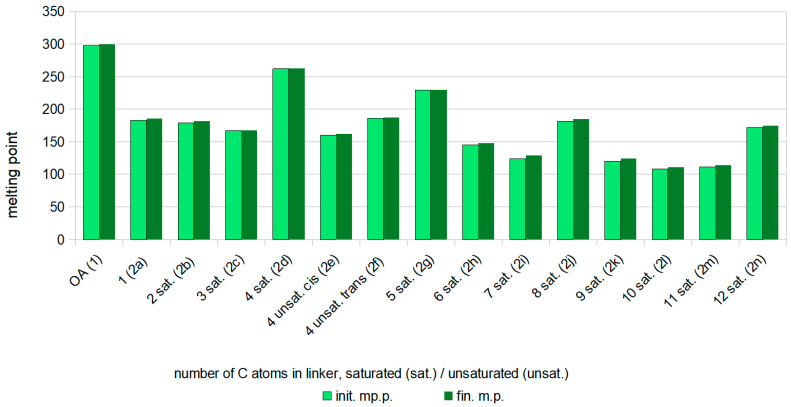
The dependency between the melting point of OADs **2a**–**2n** and the amount of carbon atoms in the linker joining two triterpene moieties. **Legend**: **OA**—oleanolic acid (reference compound); **sat.**—saturated linker; **unsat.**—unsaturated linker; **init. m.p.**—initial melting point; **fin. m.p.**—final melting point.

Figure 5 shows the relationship between the length of the linker connecting two oleanolic acid moieties (X axis) and the melting point of the purified dimer (Y axis).

The melting points (m.p.) of all OADs (**2a**–**2n**) were lower than the melting point of oleanolic acid (298–300 °C), regardless of the linker length, and were in the range of 108–230 °C; the exception was the dimer with a saturated four-carbon linker **2d**, which melted at 262–263 °C (Figure 5).

Connecting two residues of oleanolic acid with the shortest, one-carbon bridge (dimer **2a**) contributed to lowering the melting point of the product by over 100 °C (298–300 °C for oleanolic acid and 183–186 °C for dimer **2a**). As the chain length increased, the melting temperature of dimers decreased, except for compounds **2d**, **2f**, **2g**, **2j**, and **2n**, for which the melting points were higher than for the neighbouring dimers with shorter chains (**2c**, **2e**, **2i**, **2m**, respectively).

The Oleanolic Acid Dimer with the shortest, one-carbon linker (**2a**) crystallised from ethanol with water. Short and moderately long linked dimers, i.e., with two to six carbon atoms in the linker (**2b**–**2h**), including compounds with both *cis* or *trans* double bonds (**2e**, **2f**, respectively), crystallised from ethanol to form tiny sparkling crystals. As the length of the linker increased, an increase in the solubility of the dimer in ethanol was observed, and difficulty in crystal formation increased. Dimers with long linkers, from 7 to 12 carbon atoms (**2i**–**2n**, respectively), were highly soluble in ethanol, but did not crystallise, which can be explained by the influence of the alkyl chain on the susceptibility to crystallisation.

Considering the dimers with saturated linkers (**2a**–**2e**, **2g**–**2n**), it is observed that the melting point decreases with increasing chain length in the linker connecting two triterpene moieties. However, this rule does not apply to products with linkers containing 4, 8 and 12 -CH_2_ groups in the linker (i.e., compounds **2d**, **2g**, **2j**, and **2n**), whose melting points are much higher (by about 80–100 °C) than the neighbouring dimers, with shorter linkers (i.e., **2c**, **2h**, **2i**, and **2m**, respectively).

#### 3.1.3. The Polarity of OADs **2a**–**2n** Compared to Polarity of Oleanolic Acid (**1**)

The polarity of the obtained OADs **2a**–**2n** was compared with the polarity of oleanolic acid (**1**) using the TLC method.

The obtained OADs **2a**–**2n** are generally compounds with a polarity comparable to oleanolic acid (**1**) (Table 5). The subtle differences that can be observed in the chromatograms sometimes made it difficult to decide whether the dimerisation reaction was completed. An excellent way to ensure that the substrate wholly converted to the intended product was to repeat the TLC control using a different eluent and a TLC plate with a longer elution path. The most favourable differences in the R_f_ value between compounds **2a**–**2n** and oleanolic acid (**1**), and therefore the most significant differences in the position of the spots of the substrate and products, were obtained using benzene with ethyl acetate in volume ratios 1:1, 2:1, 4:1, and 9:1 (Table 5).

Comparing the R_f_ values of oleanolic acid (**1**) and its dimers **2a**–**2n** (Table 5), it can be noticed that for dimers with the shortest chains forming a linker (i.e., containing one or two methylene groups in the chain, compounds **2a** and **2b**, respectively), polarity increased with increasing chain length (in the chromatogram, the spots from these two products **2a** and **2b** were located lower than the spot from oleanolic acid, **1**). With further elongation of the linker (from 3 to 6 carbon atoms in the linker, compounds **2c**–**2h**), the R_f_ value slightly decreased, for OADs containing 7–9 carbon atoms in the chain (OADs **2i**–**2k**), the R_f_ value was comparable to the R_f_ value of oleanolic acid (**1**), and, finally, for the dimers containing the longest bridges (10–12 carbon atoms in the chain, products **2l**–**2n**, respectively), the spots were observed slightly higher than those of oleanolic acid (**1**), which means that these compounds **2l**–**2n** were slightly less polar than the mother compound (**1**). It was also observed that the polarity of dimers with four-carbon linkers containing an unsaturated bond (compounds **2e** and **2f**) was almost the same and comparable to the polarity of the analogous dimer but with a four-carbon saturated linker (dimer **2d**).

#### 3.1.4. Spectral Characterisation

The structures of the obtained OADs **2a**–**2n** were determined based on spectral data analysis. Because the molecules of OADs **2a**–**2n** are probably symmetrical, the analogous carbon atoms in both triterpene units forming the dimer are marked with the same numbers. The notation, for example, “2 × C-3” means analogous carbon atoms in a symmetric dimer.

Graphical spectra: IR, ^1^H NMR, ^13^C NMR, and DEPT for the exemplary OAD (dimer **2a**) are presented in Supplementary File S2.

Spectral Characterisation of Dimer **2a** (With One-Carbon Linker)

In the Infrared (IR) spectrum of dimer **2a**, a strong, broad band was observed, derived from OH groups, in the wavenumber (ν) range of 3556.01–3205.29 cm^−1^. In addition, a strong band for the carbonyl group was found at ν 1748.60 cm^−1^ along with a distinct band (ν 1467.16 cm^−1^) for the CO- group within the ester moiety.

In the Proton Nuclear Magnetic Resonance (^1^H NMR) spectrum of compound **2a**, three signals were present, derived from the protons characteristic for oleanane skeleton, located at carbon atom no. 3, 12, and 18. These signals, observed at the chemical shift (δ) values of 5.29, 3.21, and 2.83 ppm, respectively, were identical or similar to values of chemical shift in the ^1^H NMR spectrum of oleanolic acid (**1**) [36]. The same multiplicity characterised the signals. The methylene linker protons formed a signal at δ 5.74 ppm, observed as a singlet of 2C intensity.

Analysing the Carbon Nuclear Magnetic Resonance (^13^C NMR) spectrum of the dimer **2a** with the shortest linker and comparing it with the analogous spectrum of the mother oleanolic acid (**1**) allowed us to observe five signals characteristic for the oleanane skeleton, and one new signal was found. Of these five, four were located at the typical values of chemical shift characteristic for the oleanane system [36]. These were (I) the signal for the quaternary carbon atom, located at δ 143.7 ppm for oleanolic acid (**1**) and δ 143.3 ppm for the new derivative **2a**, assigned to the atom at position C-13; (II) a signal for the tertiary carbon atom located at δ 122.8 ppm (for the parent compound **1**) and at δ 122.6 ppm (for dimer **2a**), assigned to the C-12 atom; (III) a signal located at δ 78.9 ppm and 78.8 ppm, assigned to the C-3 carbon atom in the molecule of oleanolic acid (**1**) and derivative **2a**, respectively; (IV) a signal located at δ 46.7 ppm, both in the spectrum of the substrate (**1**) and the product (**2a**), originating from the quaternary carbon atom and assigned to the C-17 atom of oleanane skeleton. The fifth, among the signals characteristic of the oleanane skeleton, was observed at a significantly different chemical shift value, which confirms that the reaction occurred within the carboxyl group. This signal, derived from a quaternary carbon atom, was located at δ 184.1 ppm for oleanolic acid (**1**) and at δ 176.3 ppm for product **2a**.

In the ^13^C NMR spectrum of derivative **2a**, the presence of one more signal was observed, located at δ 79.3 ppm. This signal had an intensity of about half that of the other signals from the CH_2_ groups. Based on this observation, it can be concluded that the new signal (δ 79.3 ppm) has an intensity of 1C, while the remaining signals from the CH_2_ groups have an intensity of 2C. This information indicates that it was a signal derived from a methylene linker that joined two moieties of oleanolic acid (**1**) using its carboxyl group.

The Distortionless Enhancement by Polarization Transfer (DEPT) spectrum of dimer **2a** presented seven signals for primary carbon atoms, eleven for secondary carbon atoms, and five for tertiary carbon atoms.

Summary of IR Spectral Data of OADs (**2a**–**2n**)

The wavenumber values (ν) for the most important signals present in the IR spectra of OADs **2a**–**2n** are presented in Table 6. The relevant spectral data for oleanolic acid (**1**) are also given for comparison.

Analysing the IR spectra of OADs **2a**–**2n**, a strong broad absorption band was observed, located at approximately ν 3555–3205 cm^−1^ (Table 6), which originated from vibrations within the OH group. The second strong band, narrow, was observed in the IR spectra of almost all OADs (except **2a** and **2b**) at ν about 1726 or 1727 cm^−1^ and was attributed to the carbonyl group; for dimers **2a** and **2b,** this band was observed at ν 1748.60 and 1736.21 cm^−1^, respectively. The shift of the position of the discussed absorption band towards higher wavenumber values (ν 1748.60 and 1736.21 cm^−1^ for dimers **2a** and **2b**, respectively) is probably related to the presence of a short linker located near the C=O group (for all other dimers, **2c**–**2n**, the linker contained at least three carbon atoms). The third characteristic absorption band was present in the IR spectra of almost all OADs (except **2a** and **2b**) at ν around 1462 or 1463 cm^−1^ and originated from the vibrations of the C-O group. For dimers **2a** and **2b**, this absorption band was observed at slightly higher wavenumber values (1467.60 cm^−1^ and 1466.29 cm^−1^, respectively), and the reason for the position of this band shifting was also probably the presence of a short linker near the C-O- group.

Summary of ^1^H NMR Spectral Data of OADs (**2a**–**2n**)

The values of chemical shifts (δ) for the most important signals present in the ^1^H NMR spectra of OADs **2a**–**2n**, characteristic for the oleanane system and the extreme atoms of the linker connecting two triterpene residues, are presented in Table 7. The relevant spectral data for oleanolic acid (**1**) are also given for comparison.

For all OADs (compounds **2a**–**2n**), the signal from the C-12 proton was observed at chemical shift (δ) 5.28 or 5.29 ppm (rarely at δ 5.30 ppm) and always formed a triplet (t) with a coupling constant (*J*) in the range from 3.4 to 3.7 Hz (Table 7). For oleanolic acid (**1**), this signal was also observed as a triplet (t), located at δ 5.27 ppm and the coupling constant of 3.5 Hz. The signal coming from the proton at the C-3 position, to which the hydroxyl group is attached in molecules of OADs (**2a**–**2n**), was always present as a doublet of doublets (dd) in the ^1^H NMR spectra, usually observed at δ 3.21 ppm (rarely at other value of chemical shift) with coupling constants of about 11 and 4 Hz (for oleanolic acid, **1**: δ 3.21 ppm, *J* = 10.1 and 5.8 Hz). In turn, the third signal characteristic for the oleanane system, originating from the proton at the C-18 position, was generally observed at δ ~2.9 ppm and always formed a doublet of doublets (dd) with a coupling constant of about 14 and 4 Hz (for oleanolic acid, **1**: δ 2.82 ppm and *J* = 13.7 and 4.1 ppm). All listed values of chemical shifts and coupling constants are close to the corresponding characteristic values typical of oleanolic acid and its unsubstituted derivatives [36].

The signal derived from protons attached to the extreme carbon atoms within the linkers of OADs **2b**–**2n** was usually observed in the range of δ 4.00–4.63 ppm, and only for the dimer with a one-carbon linker (**2a**) at δ 5.74 ppm. This signal was most often observed in the form of a multiplet (m; for dimers **2b**, **2d**, **2i**, **2k**, **2l**, and **2n**) or a triplet (t; for dimers **2c**, **2f**, **2g**, and **2h**), and rarely as a triplet of doublets (td; for dimers **2j** and **2m**). Only for the dimer with the shortest linker was the discussed signal observed in the form of a singlet (s), while for the dimer **2e**, with a four-carbon linker containing a *cis*-unsaturated bond, the discussed signal was observed as a doublet of doublet of doublets (ddd).

Summary of ^13^C NMR Spectral Data of OADs (**2a**–**2n**)

The values of chemical shifts for the most important signals present in the ^13^C NMR spectra of OADs **2a**–**2n**, characteristic of the oleanane system and the extreme atoms of the linker connecting two triterpene residues, are presented in Table 8. The relevant spectral data for oleanolic acid (**1**) are also given for comparison.

In the ^13^C NMR spectra of OADs (**2a**–**2n**), signals derived from the characteristic carbon atoms of the oleanane system were usually observed at typical values of chemical shifts [36], namely, δ ~79 ppm (C-3), δ ~122 ppm (C-12), δ ~144 ppm (C-13), and δ ~46 ppm (C-17). Only the fifth characteristic signal, coming from the C-28 atom, was observed at lower values of chemical shifts than in the analogous spectrum of oleanolic acid (δ 181.59 ppm for mentioned acid, **1**), and for OADs **2a**–**2n**, it had a δ value of about 177 ppm. The extreme carbon atoms in the linker formed a signal observed in the range of δ 60–64 ppm, except for the derivative with a bridge containing an unsaturated bond in the *cis* system (**2e**), for which the discussed signal was observed at a significantly lower value of chemical shift, i.e., at δ 59.76 ppm. The second exception was the derivative with a one-carbon bridge (**2a**), for which the analysed signal was observed at δ 79.32 ppm. It was also observed that with the increase in the amount of methylene groups in the linker, there was usually a slight shift of the position of the analysed signal towards higher values of chemical shift, but this regularity did not apply to dimer derivatives containing 11 or more carbon atoms in a linker (the above rule was not met by the derivative **2m** with an 11-carbon bridge and the derivative **2n** with a 12-carbon bridge).

### 3.2. SAR Analysis

The probability of occurrence of a given activity is defined as P_a,_ and the probability of non-occurrence of this activity as P_i_. Both values are expressed in a range between 0 and 1. If, for a given compound, the value of P_a_ is greater than 0.700, it indicates a high probability that this compound will also show this activity in experimental studies and will prove to be a functional analogue of an existing drug. If the P_a_ value is contained in the range of 0.500–0.700, there is a significant probability that the substance will show such experimental activity but will not be structurally similar to known active substances. Where the calculated probability occurrence of a given activity is below 0.500, there is only a small chance that the analysed compound will show a specific biological activity under the conditions of the experiment. However, if the experiment confirms such action, the given compound may become a New Leading Structure [37].

A probability of occurrence of a given activity (P_a_) above 0.700 was noted for all 14 OADs **2a**–**2n** (Table 1 and Table 2). For these compounds, the program predicted 40 types of activity, with antitussive, vasodilator peripheral, and wound-healing activity shown only by dimer **2a**, with the shortest, one-carbon linker. For eight types of pharmacological activity (caspase 3 stimulator, hepatoprotectant, insulin promoter, lipid metabolism regulator, membrane integrity antagonist, oxidoreductase inhibitor, transcription factor NF kappa B stimulator, and transcription factor stimulator), the level of a given activity above 0.900 was predicted for almost all 14 OADs (**2a**–**2n**). For some predicted activities, the dimers with unsaturated linkers (**2e** and **2f**) behaved atypically—they were the only, or almost the only, out of the 14 Oleanolic Acid Dimers that showed lower activity than the other dimers (alkenylglycerophosphocholine hydrolase inhibitor, anti-inflammatory, antiprotozoal, antipruritic, diacylglycerol *O*-acyl transferase inhibitor, gastrin inhibitor, lipid peroxidase inhibitor, phospholipase inhibitor), or significantly higher (antineoplastic, antiulcerative, chemopreventive, cytoprotectant, protein phosphatase inhibitor).

Taking into account the relationship between the occurrence probability of a high level of pharmacological activity and the structure of OADs, and in particular the structure of the linker, it can be observed that the length of the linker and, in some cases, its structure (double bond in the cis/trans position) influence the chance of occurrence of a given activity (Table 1 and Table 2). Dimers with the shortest linkers (up to four atoms) can probably be highly effective caspase 3 stimulants, lipid peroxidase inhibitors, and transcription factor NF kappa B stimulants. In turn, only OADs with a four-carbon unsaturated linker (either cis or trans) can be active chemopreventive agents. Connecting two oleanolic acid residues with linkers longer than one carbon can result in obtaining substances that are highly effective insulin promoters, lipid metabolism regulators, membrane integrity antagonists, and oxidoreductase inhibitors. On the contrary, the use of dimers with linkers built of two or more carbon atoms may reduce the likelihood of OADs acting effectively as apoptosis agonists (Table 1 and Table 2).

It was observed that only for OADs containing from one to four -CH_2_- groups in the linker, as the linker lengthens, there was an increase or decrease in the level of biological activity (Table 1 and Table 2).

In summary, OADs (**2a**–**2n**) may exhibit several valuable pharmacological activities in vitro in experimental studies, with the highest levels of these activities expected from short-linker dimers containing four or fewer saturated carbon atoms.

### 3.3. Cytotoxic Activity

#### 3.3.1. MTT Assay

The half-maximal inhibitory concentration (IC_50_) value for oleanolic acid (**1**), measured for the SKRB-3, SKOV-3, PC-3, and U-87 cancer cell lines, ranged from 18 to 19 μM, and for the normal cell lines, it was approximately 25 μM (Table 3). The transformation of oleanolic acid (**1**) into its dimers (**2a**–**2n**) in almost every case (except for dimer **2l** with a ten-carbon bridge) contributed to an increase in the level of cytotoxic activity in relation to the four cancer cell lines tested. For almost all OADs **2a**–**2n**, the IC_50_ value was within the range of 1.12–10.68 μM, which means that the **2a**–**2k** and **2m**–**2n** dimers were from 2 to over 17 times more active than the parent oleanolic acid (**1**). According to the literature data [38], chemical compounds present good cytotoxicity for IC_50_ ≤ 10 µM and moderate cytotoxicity for 10 µM < IC_50_ ≤ 30 µM.

Of the 14 OADs tested, only the dimer **2l**, with a ten-carbon bridge, showed cytotoxic activity towards the four cancer cell lines used at a level similar to or only slightly higher (IC_50_ 18.37 or 18.71 μM) than the parent oleanolic acid (**1**). The remaining 13 OADs became much more active cytotoxic agents against the SKRB-3, SKOV-3, PC-3, and U-87 cancer cell lines. One of these dimers, compound **2j**, with an eight-carbon bridge, presented an IC_50_ value in the range of 9.81–10.68 μM, which means that this dimer was approximately two times more active than oleanolic acid (**1**) against all four cancer cell lines. For OADs **2b**, **2c**, **2f**, **2g**, **2k**, and **2n** (with a two-, three-, and four-carbon trans-unsaturated bridge, and a five-, six-, nine-, and twelve-carbon bridge), the IC_50_ value ranged from 4.79 to 8.22 μM, which means that these OADs were two or even three times more active against all four types of cancer cells than oleanolic acid (**1**). The next group consisted of dimers **2a** (with a one-carbon bridge), **2i** (with a seven-carbon bridge), and **2m** (with an eleven-carbon bridge), for which the IC_50_ value ranged from 3.06 to 4.58 μM, i.e., they were as much as four to six times more active than the parent compound (**1**). The two most active OADs contained a four-carbon saturated bridge (dimer **2d**) or a four-carbon unsaturated bridge with a *cis* double bond (dimer **2e**).

The second of the mentioned dimers, compound **2e**, was as much as seven to nine times more active than its parent compound (**1**), and its IC_50_ values for the SKRB-3, SKOV-3, PC-3, and U-87 lines were 2.61, 2.09, 2.27, and 2.74 μM, respectively. The compound with a saturated four-carbon bridge (**2e**) was the most active of the 14 OADs **2a**–**2n** obtained. Although no clear relationship was observed between the length of the bridge connecting two triterpene units and the level of cytotoxic activity of the dimer, taking into account the structure of compounds **2a**–**2n** and their effectiveness as cytostatic agents, it can be suspected that the high level of this activity for dimer **2d**, with a four-carbon saturated bridge, and for dimer **2e**, also with a four-carbon bridge but *cis*-unsaturated, is closely related to the structure of the bridge, and more precisely to the number of methylene groups in the bridge (dimer **2d**) or to the presence of a double bond in the *cis* system (dimer **2e**).

The IC_50_ value for normal HDF cells was 24.8 for oleanolic acid, and for OADs **2a**–**2n**, it ranged from 2.68–36.27 μM. The conclusions from the comparison of the Selectivity Index values for oleanolic acid (**1**) and its dimers **2a**–**2n** will be presented in a later chapter of this work.

#### 3.3.2. Selectivity Index

Table 4 presents values of the Selectivity Index (SI) for oleanolic acid (**1**) and OADs **2a**–**2n**, expressed as a simple ratio of IC_50_ calculated for healthy and cancer cells [39]. Evaluation of the SI value for any research on herbal drugs, isolated compounds, and chemically modified compounds of natural origin is very crucial for determining whether further work can be continued. Pena-Moran et al. state that a chemical compound is worth further testing for cytotoxic/anticancer activity if the SI value is >10 [40]. Valderrama and co-workers give a much lower SI value for substances that would become potential anticancer agents. This value must be at least 2.0 [41].

Of the 14 OADs tested (**2a**–**2n**), as many as 10 showed a Selectivity Index value higher than 1.00—only dimers **2f** (with a trans-unsaturated linker) and **2e** (with a five-carbon saturated bridge) showed low or moderate SI values. Among these ten OADs, as many as four of them had a value exceeding 2.0. According to Valderrama et al. [41], triterpenes **2c**, **2d**, **2h**, **2k**, and **2e** may be considered potential anticancer compounds.

### 3.4. Antioxidant Activity

Firstly, the structural modifications introduced in the synthesised derivatives **2a**–**2n** represent a strategic approach to enhance the antioxidant potential of oleanolic acid (mother compound, **1**). Structural alterations, such as functional group substitutions or side chain modifications, can influence the compound’s ability to scavenge free radicals and mitigate oxidative stress. Researchers aim to elucidate the structural features critical for optimal antioxidant efficacy by systematically varying the molecular structure.

The notable variations in antioxidant activity observed among the derivatives underscore the importance of structure–activity relationships in rational drug design. OADs **2a** (with a one-carbon linker), **2b** (with a two-carbon linker), and **2f** (with a trans-unsaturated four-carbon linker) emerge as up-and-coming candidates, exhibiting significantly higher Trolox equivalents than OA (**1**). This suggests that specific structural modifications have enhanced radical-scavenging capabilities, surpassing the parent compound’s. Further analysis of the molecular interactions underlying these enhancements may elucidate key determinants of antioxidant potency and guide the design of future derivatives with improved bioactivity [42].

Moreover, the findings underscore the potential of synthesised derivatives of OA as valuable assets in pharmaceutical and nutraceutical applications. Compounds exhibiting superior antioxidant activity hold promise for developing therapeutic agents targeting oxidative stress-related disorders, such as cardiovascular diseases, neurodegenerative disorders, and cancer [43,44]. Additionally, identifying derivatives with enhanced bioactivity may pave the way for formulating novel antioxidant supplements with improved efficacy and bioavailability.

However, it is essential to acknowledge certain limitations and complexities inherent in antioxidant research. The DPPH assay, while widely employed for its simplicity and reliability, represents only one facet of antioxidant activity and may not fully capture the diverse mechanisms underlying cellular antioxidant defence. Therefore, complementary assays and in vivo studies are warranted to validate the observed findings and elucidate the broader physiological relevance of the synthesised derivatives.

## 4. Materials and Methods

### 4.1. General Information

Synthesis: Materials applied for syntheses are presented elsewhere [24].

Cytotoxic activity: Human cancer cells SKBR-3 (human breast cancer cell line) and SKOV-3 (ovarian cancer cell line) were cultured in McCoy’s Modified Medium. Human cancer cells PC-3 (human prostate cancer cell line) were cultured in an F-12K medium. The Human Dermal Fibroblast (HDF) cell line was cultured in Fibroblast Basal Medium. The U-87 cell line was cultured in EMEM medium. Each medium was supplemented with 10% foetal bovine serum, 1% L- glutamine, and 1% penicillin/streptomycin solution. The cell lines were kept in an incubator at 37 °C. All the cell lines and mediums were obtained from the American Type Culture Collection (ATCC) supplied by LGC-Standards (Lomianki, Poland).

Antioxidant activity: Materials applied for the DPPH assay are presented elsewhere [45].

### 4.2. Preparation of OADs

#### General Method for Dimerisation of Oleanolic Acid (1)

Natrium carbonate (277 mg, 2.0 mmol) was added to a saturated solution of oleanolic acid (456 mg, 1.0 mmol), which was stirred and heated at 80 °C. The heating and stirring was continued for 30 min, and next, α,ω-dibromoalkane/α,ω-dibromoalkene (0.5 mmol) was added and stirred at 80 °C for the next 30 min. The mixture after reaction was cooled, poured into 5 volumes of water and slightly acidified with diluted HCl. The resulting precipitate was filtered off, washed with water to neutralise the pH of the filtrate, dried and crystallised from ethanol or ethanol with water, or re-precipitated with water from an ethanolic solution.

Spectral characteristics contain only the signals most characteristic for the molecules of the obtained compounds.

**Dimer 2a**: C_61_H_96_O_6_; mol. mass: 925.41; yield: 475 mg (93.7%); mp. 183–185 °C (EtOH + H_2_O); **IR** (ν, cm^−1^): 3556.01–3205.29 (OH), 1748.60 (C=O), 1467.16 (C-O-); **^1^H NMR** (δ, ppm): 5.74 (2H, s, -O-CH_2_-O-), 5.29 (2H, t, *J* = 3.7 Hz, 2 × C_12_-H), 3.72 (weak, q, *J* = 7.0, CH_3_-CH_2_OH), 3.21 (2H, dd, *J* = 10.4 and 5.0 Hz, 2 × C_3_-H_α_), 2.83 (2H, dd, *J* = 13.7 and 4.8 Hz, 2 × C_18_-H_β_), 1.24 (weak, t, *J* = 8.5 Hz, CH_3_-CH_2_OH), 1.13, 0.99, 0.91 × 2, 0.90, 0.78, 0.75; (5 × 6H + 1 × 12H, 6 × s, 14 × CH_3_ group); **^13^C NMR** (δ, ppm): 176.30 (2 × C_q_, 2 × C-28), 143.29 (2 × C_q_, 2 × C-13), 122.58 (2 × CH, 2 × C-12), 79.32 (CH_2_, -O-CH_2_-O-), 78.95 (2 × CH, 2 × C-3), 58.39 (weak CH_2_, CH_3_-CH_2_OH), 46.71 (2 × C_q_, 2 × C-17), 18.39 (weak CH_3_, CH_3_-CH_2_OH); **DEPT:** CH_3_: 7 × 2 CH_3_ (7 signals, 14 CH_3_ groups), CH_2_: 10 × 2 CH_2_ + 1 × 1 CH_2_ (11 signals, 21 CH_2_ groups), CH: 5 × 2 CH (5 signals, 10 CH groups).

**Dimer 2b**: C_62_H_100_O_6_; mol. mass: 941.47; yield: 440 mg (=93.8%); m.p. 179–182 °C (EtOH); **IR** (ν, cm^−1^): 3553.84–3203.87 (OH), 1736.21 (C=O), 1466.29 (C-O-); **^1^H NMR** (δ, ppm): 5.29 (2H, t, *J* = 3.5 Hz, 2 × C_12_-H), 4.30–4.10 (4H, m, -O-CH_2_-CH_2_-O-), 3.71 (weak, dd, *J* = 14.1 and 7.0 Hz, CH_3_-CH_2_OH), 3.21 (2H, dd, *J* = 10.3 and 4.9 Hz, 2 × C_3_-H_α_), 2.86 (2H, dd, *J* = 13.6 and 3.7 Hz, 2 × C_18_-H_β_), 1.23 (weak, t, *J* = 8.5 Hz, CH_3_-CH_2_OH), 1.14, 0.99, 0.92, 0.91 × 2, 0.78, 0.73 (5 × 6H + 1 × 12H, 6 × s, 14 × CH_3_ group); **^13^C NMR** (δ, ppm): 177.44 (2 × C_q_, 2 × C-28), 143.51 (2 × C_q_, 2 × C-13), 122.46 (2 × CH, 2 × C-12), 78.92 (2 × CH, 2 × C-3), 62.15 (2 × CH_2_, -O-CH_2_-CH_2_-O-), 58.37 (weak, CH_2_, CH_3_-CH_2_OH), 46.63 (2 × C_q_, 2 × C-17), 18.38 (weak, CH_3_, CH_3_-CH_2_OH); **DEPT**: CH_3_: 7 × 2 CH_3_ (7 signals, 14 CH_3_ groups), CH_2_: 11 × 2 CH_2_ (11 signals, 22 CH_2_ groups), CH: 5 × 2 CH (5 signals, 10 CH groups).

**Dimer 2c**: C_63_H_100_O_6_; mol. mass: 955.50; yield: 450 mg (=94.5%); m.p. 167–168 °C (EtOH); **IR** (ν, cm^−1^): 3553.84–3203.87 (OH), 1728.25 (C=O), 1462.19 (C-O-); **^1^H NMR** (δ, ppm): 5.28 (2H, t, *J* = 3.3 Hz, 2 × C_12_-H), 4.09 (4H, t, *J* = 6.2 Hz, -O-CH_2_-CH_2_-CH_2_-O-), 3.71 (weak, dd, *J* = 14.0 and 7.1 Hz, CH_3_-CH_2_OH), 3.21 (2H, dd, *J* = 11.1 and 4.7 Hz, 2 × C_3_-H_α_), 2.85 (2H, dd, *J* = 13.7 and 3.9 Hz, 2 × C_18_-H_β_), 1.99–1.84 (6H, m, -O-CH_2_-CH_2_-CH_2_-O- and 2 × C_11_-H_2_), 1.23 (weak, t, *J* = 6.9 Hz, CH_3_-CH_2_OH), 1.13, 0.98, 0.92, 0.90 × 2, 0.78, 0.72 (5 × 6H + 1 × 12H, 6 × s, 14 × CH_3_ group); **^13^C NMR** (δ, ppm): 177.34 (2 × C_q_, 2 × C-28), 143.61 (2 × C_q_, 2 × C-13), 122.20 (2 × CH, 2 × C-12), 78.87 (2 × CH, 2 × C-3), 60.76 (2 × CH_2_, -O-CH_2_-CH_2_-CH_2_-O-), 58.11 (weak, CH_2_, CH_3_-CH_2_OH), 46.65 (2 × C_q_, 2 × C-17), 28.04 (CH_2_, -O-CH_2_-CH_2_-CH_2_-O-), 18.17 (weak, CH_3_, CH**_3_**-CH_2_OH); **DEPT**: CH_3_: 7 × 2 CH_3_ (7 signals, 14 CH_3_ groups), CH_2_: 11 × 2 CH_2_ + 1 × 1 CH_2_ (12 signals, 23 CH_2_ groups), CH: 5 × 2 CH (5 signals, 10 CH groups).

**Dimer 2d**: C_64_H_104_O_6_; mol. mass: 969.53; yield: 461 mg (=95.2%); m.p. 262–263 °C (EtOH); **IR** (ν, cm^−1^): 3596–3201.82 (OH), 1728.25 (C=O), 1462.19 (C-O-); **^1^H NMR** (δ, ppm): 5.28 (2H, t, *J* = 3.7 Hz, 2 × C_12_-H), 4.30–4.08 (4H, m, -O-CH_2_-CH_2_-CH_2_-CH_2_-O-), 3.71 (weak, dd, *J* = 14.0 and 7.0 Hz, CH_3_-CH_2_OH), 3.21 (2H, dd, *J* = 10.4 and 4.8 Hz, 2 × C_3_-H_α_), 2.86 (2H, dd, *J* = 13.7 and 4.8 Hz, C_18_-H_β_), 1.86 (4H, d, *J* = 3.20 Hz, -O-CH_2_**-**CH_2_-CH_2_-CH_2_-O-), 1.24 (weak, t, *J* = 7.0 Hz, CH_3_-CH_2_OH), 1.13, 0.99, 0.92, 0.90 × 2, 0.78, 0.73 (5 × 6H + 1 × 12H, 6 × s, 14 × CH_3_ group); **^13^C NMR** (δ, ppm): 177.62 (2 × C_q_, 2 × C-28), 143.74 (2 × C_q_, 2 × C-13), 122.36 (2 × CH, 2 × C-12), 78.91 (2 × CH, 2 × C-3), 63.65 (2 × CH_2_, -O-CH_2_-CH_2_-CH_2_-CH_2_-O-), 58.11 (weak, CH_2_, CH_3_-CH_2_OH,), 46.66 (2 × C_q_, 2 × C-17), 25.41 (2 × CH_2_, -O-CH_2_**-**CH_2_-CH_2_-CH_2_-O-), 18.17 (weak, CH_3_, CH_3_-CH_2_OH); **DEPT**: CH_3_: 7 × 2 CH_3_ (7 signals, 14 CH_3_ groups), CH_2_: 12 × 2 CH_2 (_12 signals, 24 CH_2_ groups), CH: 5 × 2 CH (5 signals, 10 CH groups).

**Dimer 2e**: C_64_H_102_O_6_; mol. mass: 967.51; yield: 449 mg (=92.8%); m.p. 160–162 °C (EtOH); **IR** (ν, cm^−1^): 3551.81–3207.99 (OH), 1726.31 (C=O), 1462.47 (C-O-); **^1^H NMR** (δ, ppm): 5.70 (2H, t, *J* = 4.1 Hz, -O-CH_2_-CH=CH-CH_2_-O-), 5.28 (2H, t, *J* = 3.5 Hz, 2 × C_12_-H), 4.63 (4H, ddd, *J* = 17.8, 13.0 and 4.3 Hz, -O-CH_2_-CH=CH-CH_2_-O-), 3.21 (2H, dd, *J* = 11.1 and 4.8 Hz, 2 × C_3_-H_α_), 2.85 (2H, dd, *J* = 13.8 and 4.0 Hz, 2 × C_18_-H_β_), 1.13, 0.99, 0.92, 0.91, 0.90, 0.78, 0.72 (7 × 6H, 7 × s, 14 CH_3_ groups); **^13^C NMR** (δ, ppm): δ 177.29 (2 × C_q_, 2 × C-28), 143.58 (2 × C_q_, 2 × C-13), 128.21 (2 × CH, -O-CH_2_-CH=CH-CH_2_-O-), 122.38 (2 × CH, 2 × C-12), 78.93 (2 × CH, 2 × C-3), 59.76 (2 × CH_2_, -O-CH_2_-CH=CH-CH_2_-O-), 46.61 (2 × C_q_, 2 × C-17); **DEPT**: CH_3_: 7 × 2 CH_3_ (7 signals, 14 CH_3_ groups), CH_2_: 11 × 2 CH_2_ (11 signals, 22 CH_2_ groups), CH: 6 × 2 CH (6 signals, 12 CH groups).

**Dimer 2f**: C_64_H_102_O_6_; mol. mass: 967.51; yield: 446 mg (=92.2%); m.p. 186–187 °C (EtOH); **IR** (ν, cm^−1^): 3550.46–32208.67 (OH), 1726.32 (C=O), 1462.54 (C-O-); **^1^H NMR** (δ, ppm): 5.87 (2H, t, *J* = 2.8 Hz, -O-CH_2_-CH=CH-CH_2_-O-), 5.30 (2H, t, *J* = 3.4 Hz, 2 × C_12_-H), 4.45 (4H, t, *J* = 13.9 Hz, -O-CH_2_-CH=CH-CH_2_-O-), 3.21 (2H, dd, *J* = 11.5 and 3.9 Hz, C_3_-H_α_), 2.77 (2H, dd, *J* = 14.1 and 4.1 Hz, C_18_-H_β_), 1.14, 0.99, 0.93, 0.91 × 2, 0.78, 0.72 (5 × 6H + 1 × 12H, 6 × s, 14 × CH_3_ group); **^13^C NMR** (δ, ppm): 177.33 (2 × C_q_, 2 × C-28), 143.67 (2 × C_q_, 2 × C-13), 127.94 (2 × CH, -O-CH_2_-CH=CH-CH_2_-O-), 122.39 (2 × CH, 2 × C-12), 78.89 (2 × CH, 2 × C-3), 63.72 (2 CH_2_, -O-CH_2_-CH=CH-CH_2_-O-), 46.76 (2 × C_q_, 2 × C-17); **DEPT**: CH_3_: 7 × 2 CH_3_ (7 signals, 14 CH_3_ groups), CH_2_: 11 × 2 CH_2_, (11 signals, 22 CH_2_ groups), CH: 6 × 2 CH (6 signals, 12 CH groups).

**Dimer 2g**: C_65_H_106_O_6_; mol. mass: 983.55; yield: 933 mg (=94.9%); m.p. 229–230 °C (EtOH); **IR** (ν, cm^−1^): 3554.18–3203.87 (OH), 1726.32 (C=O), 1462.19 (C-O-); **^1^H NMR** (δ, ppm): δ 5.29 (2H, t, *J* = 3.6 Hz, 2 × C_12_-H), 4.07 (4H, t, *J* = 6.5 Hz, -O-CH_2_-CH_2_-CH_2_-CH_2_-CH_2_-O-), 3.21 (2H, dd, *J* = 11.2 and 4.6 Hz, 2 × C_3_-H_α_), 2.86 (2H, dd, *J* = 13.6 and 4.1 Hz, 2 × C_18_-H_β_), 1.13, 0.99, 0.92, 0.90 × 2, 0.78 and 0.73 (5 × 6H + 1 × 12H, 6 × s, 14 × CH_3_ group); **^13^C NMR** (δ, ppm): 177.64 (2 × C_q_, 2 × C-28), 143.70 (2 × C_q_, 2 × C-13), 122.29 (2 × CH, 2 × C-12), 78.89 (2 × CH, 2 × C-3), 63.94 (2 × CH_2_, -O-CH_2_-CH_2_-CH_2_-CH_2_-CH_2_-O-), 46.59 (2 × C_q_, 2 × C-17), 28.21 (2 × CH_2_, -O-CH_2_**-**CH_2_-CH_2_-CH_2_-CH_2_-O-); 22.95 (CH_2_, -O-CH_2_**-**CH_2_-CH_2_-CH_2_-CH_2_-O-); **DEPT**: CH_3_: 7 × 2 CH_3_ (7 signals, 14 CH_3_ groups), CH_2_: 12 × 2 CH_2_ + 1 × 1 CH_2_ (13 signals, 25 CH_2_ groups), CH: 5 × 2 CH (5 signals, 10 CH groups).

**Dimer 2h**: C_66_H_108_O_6_; mol. mass: 997.58; yield: 972 mg (=97.4%); m.p. 145–148 °C (EtOH); **IR** (ν, cm^−1^): 3557.89–3208.67 (OH), 1726.32 (C=O), 1462.54 (C-O-); **^1^H NMR** (δ, ppm): 5.28 (2H, t, *J* = 3.6 Hz, 2 × C_12_-H), 4.01 (4H, t, *J* = 6.5 Hz, 2 × -O-CH_2_-CH_2_-CH_2_-CH_2_-CH_2_-CH_2_-O-), 3.72 (weak, dd, *J* = 14.1 and 7.1 Hz, CH_3_-CH_2_OH), 3.21 (2H, dd, *J* = 10.5 and 5.0 Hz, C_3_-H_α_), 2.87 (2H, dd, *J* = 13.7 and 3.9 Hz, C_18_-H_β_), 1.24 (weak, t, *J* = 7.0 Hz, CH_3_-CH_2_OH); 1.13, 0.99, 0.92, 0.90 × 2, 0.78, 0.73 (5 × 6H + 1 × 12H, 6 × s, 14 × CH_3_ group); **^13^C NMR** (δ, ppm): 177.69 (2 × C_q_, 2 × C-28), 144.01 (2 × C_q_, 2 × C-13), 122.29 (2 × CH, 2 × C-12), 78.82 (2 × CH, 2 × C-3), 64.15 (2 × CH_2_, -O-CH_2-_CH_2_-CH_2_-CH_2_-CH_2_-CH_2_-O-), 58.36 (weak, CH_2_, CH_3_-CH_2_OH), 46.63 (2 × C_q_, 2 × C-17), 28.55 (2 × CH_2_, -O-CH_2_-CH_2_-CH_2_-CH_2_-CH_2_-CH_2_-O-), 25.83 (2 × CH_2_, -O-CH_2_-CH_2_-CH_2_-CH_2_-CH_2_-CH_2_-O-), 18.30 (weak, CH_3_, CH_3_-CH_2_OH); **DEPT**: CH_3_: 7 × 2 CH_3_ (7 signals, 14 CH_3_ groups), CH_2_: 13 × 2 CH_2_ (13 signals, 26 CH_2_ groups), CH: 5 × 2 CH (5 signals, 10 CH groups).

**Dimer 2i**: C_67_H_110_O_6_; mol. mass: 1011.61; yield: 939 mg (=92.8%); m.p. 124–129 °C (precip. with H_2_O from EtOH sol.); **R** (ν, cm^−1^): 3557.89–3208.67 (OH), 1726.21 (C=O), 1463.83 (C-O-); **^1^H NMR (**δ, ppm): 5.29 (2H, t, *J* = 3.5 Hz, 2 × C_12_-H), 4.10–3.94 (4H, m, -O-CH_2_-CH_2_-CH_2_-CH_2_-CH_2_-CH_2_-CH_2_-O-), 3.22 (2H, dd, *J* = 11.3 and 4.3 Hz, 2 × C_3_-H_α_), 2.87 (2H, dd, *J* = 14.0 and 4.7 Hz, 2 × C_18_-H_β_), 1.14, 1.00, 0.93, 0.91 × 2, 0.79 and 0.74 (5 × 6H + 1 × 12H, 6 × s, 14 × CH_3_ group); **^13^C NMR (**δ, ppm): 177.64 (2 × C_q_, 2 × C-28), 143.57 (2 × C_q_, 2 × C-13), 122.21 (2 × CH, 2 × C-12), 78.97 (2 × CH, 2 × C-3), 64.05 (2 × CH_2_, -O-CH_2_-CH_2_-CH_2_-CH_2_-CH_2_-CH_2_-CH_2_-O-), 46.67 (2 × C_q_, 2 × C-17), 28.85 (1 × CH_2_, -O-CH_2_-CH_2_-CH_2_-CH_2_-CH_2_-CH_2_-CH_2_-O-), 28.55 (2 × CH_2_, O-CH_2_**-**CH_2_-CH_2_-CH_2_-CH_2_-CH_2_-CH_2_-O-), 28.12 (2 × CH_2_, -O-CH_2_**-**CH_2_-CH_2_-CH_2_-CH_2_-CH_2_-CH_2_-O-); **DEPT**: CH_3_: 7 × 2 CH_3_ (7 signals, 14 CH_3_ groups), CH_2_: 13 × 2 CH_2_ + 1 × 1 CH_2_ (14 signals, 27 CH_2_ groups), CH: 5 × 2CH (5 signals, 10 CH groups).

**Dimer 2j**: C_68_H_112_O_6_; mol. mass: 1025.63; yield: 991 mg (=96.6%); m.p. 181–185 °C (precip. with H_2_O from EtOH sol.); **IR** (ν, cm^−1^): 3557.98–3204.95 (OH), 1728.25 (C=O), 1462.54 (C-O-); **^1^H NMR** (δ, ppm): 5.29 (2H, t, *J* = 3.7 Hz, 2 × C_12_-H), 4.02 (4H, td, *J* = 6.5 and 2.0 Hz, -O-CH_2_-CH_2_-CH_2_-CH_2_-CH_2_-CH_2_-CH_2_-CH_2_-CH_2_-O-), 3.25 (2H, dd, *J* = 10.8 and 4.6 Hz, 2 × C_3_-H_α_), 2.89 (2H, dd, *J* = 13.7 and 3.8 Hz, 2 × C_18_-H_β_), 1.15, 1.01, 0.94, 0.92, 0.92, 0.80, 0.75 (7 × 6H, 7 × s, 14 × CH_3_ groups); **^13^C NMR (**δ, ppm): 177.77 (2 × C_q_, 2 × C-28), 143.86 (2 × C_q_, 2 × C-13), 122.34 (2 × CH, 2 × C-12), 79.01 (2 × CH, 2 × C-3), 64.23 (2 × CH_2_, -O-CH_2_-CH_2_-CH_2_-CH_2_-CH_2_-CH_2_-CH_2_-CH_2_-O-), 46.68 (2 × C_q_, 2 × C-17), 29.20 (2 × CH_2_, -O-CH_2_-CH_2_-CH_2_-CH_2_-CH_2_-CH_2_-CH_2_-CH_2_-O-), 28.61 (2 × CH_2_, -O-CH_2_-CH_2_-CH_2_-CH_2_-CH_2_-CH_2_-CH_2_-CH_2_-O-), 26.04 (2 × CH_2_, -O-CH_2_-CH_2_-CH_2_-CH_2_-CH_2_-CH_2_-CH_2_-CH_2_-O-); **DEPT**: CH_3_: 7 × 2 CH_3_ (7 signals, 14 CH_3_ groups), CH_2_: 14 × 2 CH_2_ (14 signals, 28 CH_2_ groups), CH: 5 × 2 CH (5 signals, 10 CH groups).

**Dimer 2k**: C_69_H_114_O_6_; mol. mass: 1039.66; yield: 998 mg (=96.0%); m.p. 120–124 °C (precip. with H_2_O from EtOH sol.); **IR** (ν, cm^−1^): 3553.84–3201.24 (OH), 1726.16 (C=O), 1462.19 (C-O-); **^1^H NMR** (δ, ppm): 5.29 (2H, t, *J* = 3.2 Hz, 2 × C_12_-H), 4.07–3.95 (2H, m, (CH_2_, -O-CH_2_-CH_2_-CH_2_-CH_2_-CH_2_-CH_2_-CH_2_-CH_2_-CH_2_-O-), 3.22 (2H, dd, *J* = 10.9 and 4.6 Hz, 2 × C_3_-H_α_), 2.87 (2H, dd, *J* = 13.9 and 3.7 Hz, 2 × C_18_-H_β_), 1.14, 0.99, 0.93, 0.91, 0.78 I 0.74 (7 × 6H, 7 × s, 14 × CH_3_ groups; **^13^C NMR** (δ, ppm): 177.76 (2 × C_q_, 2 × C-28), 143.82 (2 × C_q_, 2 × C-13), 122.32 (2 × CH, 2 × C-12), 78.96 (2 × CH, 2 × C-3), 64.24 (2 × CH_2_, -O-CH_2_-CH_2_-CH_2_-CH_2_-CH_2_-CH_2_-CH_2_-CH_2_-CH_2_-O-), 46.66 (2 × C_q_, 2 × C-17), 29.53 (2 × CH_2_, -O-CH_2_-CH_2_-CH_2_-CH_2_-CH_2_-CH_2_-CH_2_-CH_2_-CH_2_-O-), 29.14 (2 × CH_2_, -O-CH_2_-CH_2_-CH_2_-CH_2_-CH_2_-CH_2_-CH_2_-CH_2_-CH_2_-O-), 28.61 (2 × CH_2_, -O-CH_2_-CH_2_-CH_2_-CH_2_-CH_2_-CH_2_-CH_2_-CH_2_-CH_2_-O-), 26.06 (2 × CH_2_, -O-CH_2_-CH_2_-CH_2_-CH_2_-CH_2_-CH_2_-CH_2_-CH_2_-CH_2_-O-); **DEPT**: CH_3_: 7 × 2 CH_3_ (7 signals, 14 CH_3_ groups), CH_2_: 14 × 2 CH_2_ + 1 × 1 CH_2_ (15 signals, 29 CH_2_ groups), CH: 5 × 2 CH (5 signals, 10 CH groups).

**Dimer 2l**: C_70_H_116_O_6_; mol. mass: 1053.69; yield: 1011 mg (= 96.0%); m.p. 108–111 °C (precip. with H_2_O from EtOH sol.); **IR** (ν, cm-^1^): 3554.18–3201.24 (OH), 1726.32 (C=O), 1462.25 (C-O-); **^1^H NMR (**δ, ppm): 5.30 (2H, t, *J* = 3.4 Hz, 2 × C_12_-H), 4.10–3.93 (4H, m, -O-CH_2_-CH_2_-CH_2_-CH_2_-CH_2_-CH_2_-CH_2_-CH_2_-CH_2_-CH_2_-O-), 3.22 (2H, dd, *J* = 11.2 and 4.0 Hz, 2 × C_3_-H_α_), 2.88 (2H, dd, *J* = 13.8 and 4.1 Hz, 2 × C_18_-H_β_), 1.14, 1.00, 0.94, 0.92, 0.91, 0.79, 0.75 (7 × 6H, 7 × s, 14 × CH_3_ groups); **^13^C NMR** (δ, ppm): 177.77 (2 × C_q_, 2 × C-28), 143.83 (2 × C_q_, 2 × C-13), 122.32 (2 × CH, 2 × C-12), 78.99 (2 × CH, 2 × C-3), 64.25 (2 × CH_2_, -O-CH_2_-CH_2_-CH_2_-CH_2_-CH_2_-CH_2_-CH_2_-CH_2_-CH_2_-CH_2_-O-), 46.66 (2 × C_q_, 2 × C-17), 29.55 (2 × CH_2_, -O-CH_2_-CH_2_-CH_2_-CH_2_-CH_2_-CH_2_-CH_2_-CH_2_-CH_2_-CH_2_-O-), 29.24 (2 × CH_2_, -O-CH_2_-CH_2_-CH_2_-CH_2_-CH_2_-CH_2_-CH_2_-CH_2_-CH_2_-CH_2_-O-), 28.63 (2 × CH_2_, -O-CH_2_-CH_2_-CH_2_-CH_2_-CH_2_-CH_2_-CH_2_-CH_2_-CH_2_-CH_2_-O-), 26.10 (2 × CH_2_, -O-CH_2_-CH_2_-CH_2_-CH_2_-CH_2_-CH_2_-CH_2_-CH_2_-CH_2_-CH_2_-O-); **DEPT**: CH_3_: 7 × 2 CH_3_ (7 signals, 14 CH_3_ groups), CH_2_: 15 × 2 CH_2_ (15 signals, 30 CH_2_ groups), CH: 5 × 2 CH (5 signals, 10 CH groups).

**Dimer 2m**: C_71_H_116_O_6_; mol. mass: 1064.88; yield: 982 mg (=92.2%); m.p. 100–105 °C (precip. with H_2_O from EtOH sol.); **IR** (ν, cm^−1^): 3554.02–3202.91 (OH), 1726.34 (C=O), 1462.89 (C-O-); **^1^H NMR** (δ, ppm): 5.28 (2H, t, *J* = 3.4 Hz, 2 × C_12_-H), 4.00 (4H, td, *J* = 6.4 and 3.1 Hz, -O-CH_2_-CH_2_-CH_2_-CH_2_-CH_2_-CH_2_-CH_2_-CH_2_-CH_2_-CH_2_-CH_2_-O-), 3.21 (2H, dd, *J* = 11.0 and 4.6 Hz, 2 × C_3_-H_α_), 2.87 (2H, dd, *J* = 13.8 and 4.1 Hz, 2 × C_18_-H_β_), 1.13, 0.99, 0.92, 0.90 × 2, 0.78, 0.73 (5 × 6H + 1 × 12H, 6 × s, 14 CH_3_ groups); **^13^C NMR** (δ, ppm): 177.74 (2 × C_q_, 2 × C-28), 143.77 (2 × C_q_, 2 × C-13), 122.24 (2 × CH, 2 × C-12), 78.91 (2 × CH, 2 × C-3), 64.21 (2 × CH_2_, -O-CH_2_-CH_2_-CH_2_-CH_2_-CH_2_-CH_2_-CH_2_-CH_2_-CH_2_-CH_2_-CH_2_-O-), 46.62 (2 × C_q_, 2 × C-17), 29.60 (2 × CH_2_, -O-CH_2_-CH_2_-CH_2_-CH_2_-CH_2_-CH_2_-CH_2_-CH_2_-CH_2_-CH_2_-CH_2_-O-), 29.51 (1 × CH_2_, -O-CH_2_-CH_2_-CH_2_-CH_2_-CH_2_-CH_2_-CH_2_-CH_2_-CH_2_-CH_2_-CH_2_-O-), 29.20 (2 × CH_2_, -O-CH_2_-CH_2_-CH_2_-CH_2_-CH_2_-CH_2_-CH_2_-CH_2_-CH_2_-CH_2_-CH_2_-O-), 28.56 (2 × CH_2_, -O-CH_2_-CH_2_-CH_2_-CH_2_-CH_2_-CH_2_-CH_2_-CH_2_-CH_2_-CH_2_-CH_2_-O-), 26.05 (2 × CH_2_, -O-CH_2_-CH_2_-CH_2_-CH_2_-CH_2_-CH_2_-CH_2_-CH_2_-CH_2_-CH_2_-CH_2_-O-); **DEPT**: CH_3_: 7 × 2 CH_3_ (7 signals, 14 CH_3_ groups), CH_2_: 15 × 2 CH_2_ + 1 × 1 CH_2_ (16 signals, 31 CH_2_ groups), CH: 5 × 2 CH (5 signals, 10 CH groups).

**Dimer 2n**: C_72_H_118_O_6_; mol. mass: 1078.89; yield: 1034 mg (=95.9%); m.p. 172–175 °C (precip. with H_2_O from EtOH sol.); **IR** (ν, cm^−1^): 3553.93–3202.75 (OH), 1726.77 (C=O), 1463.01 (C-O-); **^1^H NMR (**δ, ppm): 5.28 (2H, t, *J* = 3.5 Hz, 2 × C_12_-H), 4.06–3.94 (4H, m, -O-CH_2_-CH_2_-CH_2_-CH_2_-CH_2_-CH_2_-CH_2_-CH_2_-CH_2_-CH_2_-CH_2_-CH_2_-O-), 3.21 (2H, dd, *J* = 11.0 and 4.6 Hz, 2 × C_3_-H_α_), 2.87 (2H, dd, *J* = 14.3 and 3.7 Hz, 2 × C_18_-H_β_), 1.27; 1.13; 0.99; 0.92; 0.90; 0.78; 0.73 (7 × 6H, 7 × s, 14 CH_3_ groups); **^13^C NMR** (δ, ppm): 177.72 (2 × C_q_, 2 × C-28), 143.75 (2 × C_q_, 2 × C-13), 122.25 (2 × CH, 2 × C-12), 78.89 (2 × CH, 2 × C-3), 64.20 (2 × CH_2_, -O-CH_2_-CH_2_-CH_2_-CH_2_-CH_2_-CH_2_-CH_2_-CH_2_-CH_2_-CH_2_-CH_2_-CH_2_-O-), 46.45 (2 × C_q_, 2 × C-17), 29.60 (2 × CH_2_, -O-CH_2_-CH_2_-CH_2_-CH_2_-CH_2_-CH_2_-CH_2_-CH_2_-CH_2_-CH_2_-CH_2_-CH_2_-O-), 29.59 (2 × CH_2_, -O-CH_2_-CH_2_-CH_2_-CH_2_-CH_2_-CH_2_-CH_2_-CH_2_-CH_2_-CH_2_-CH_2_-CH_2_-O-), 29.20 (2 × CH_2_, -O-CH_2_-CH_2_-CH_2_-CH_2_-CH_2_-CH_2_-CH_2_-CH_2_-CH_2_-CH_2_-CH_2_-CH_2_-O-), 28.56 (2 × CH_2_, -O-CH_2_-CH_2_-CH_2_-CH_2_-CH_2_-CH_2_-CH_2_-CH_2_-CH_2_-CH_2_-CH_2_-CH_2_-O-), 28.06 (2 × CH_2_, -O-CH_2_-CH_2_-CH_2_-CH_2_-CH_2_-CH_2_-CH_2_-CH_2_-CH_2_-CH_2_-CH_2_-CH_2_-O-); **DEPT**: CH_3_: 7 × 2 CH_3_ (7 signals, 14 CH_3_ groups), CH_2_: 16 × 2 CH_2_ (16 signals, 32 CH_2_ groups), CH: 5 × 2 CH (5 signals, 10 CH groups).

### 4.3. Polarity of OADs (***2a***–***2n***)

The polarity of the obtained OADs (**2a**–**2n**) was compared to that of the parent compound (**1**) and assessed by the High-Performance Thin-Layer Chromatography (HP TLC) method based on the R_f_ value.

The HP-TLC was carried out on aluminium-backed plates precoated with silica gel 60 F254 (#1.05554, Merck, Darmstadt, Germany). Dichloromethane solutions (0.5%, *w*/*v*) of the compounds **2a**–**2n** were applied (2 μL) on the starting line. Plates (10 × 10 cm) were developed at 22 ± 1 °C in a horizontal chamber previously saturated for 10 min with a vapour of a mobile phase. The mobile phase was a mixture of benzene with ethyl acetate in different volume ratios (1:1, 2:1, 4:1, 9:1, 15:1, or 25:1). After plate development and drying, spots were visualised on the plates by spraying them with 20% sulfuric acid solution in ethanol and heating at 105–110 °C for 5 min. Colour spots appeared on the white background of the plates. The chromatograms were run in duplicate and the average value of R_f_ was calculated.

### 4.4. SAR Study

The SAR study was conducted as described earlier [24].

### 4.5. MTT Assay

The MTT assay was conducted as described earlier [24].

### 4.6. Antioxidant Activity

The 2,2-diphenyl-1-picrylhydrazyl (DPPH) assay was performed according to Garbiec et al. with modifications [45]. Briefly, 25 μL of the sample at a concentration of 1.0 mg/mL dissolved in dimethyl sulfoxide (DMSO) was mixed with 175 μL of DPPH solution (3.9 mg in 50 mL of methanol). The reaction mixture was shaken and incubated in the dark at room temperature for 30 min. The control sample contained 25 μL of DMSO and 175 μL of DPPH solution. Absorbance was measured at 517 nm. The inhibition of the DPPH radical by the sample was calculated according to the following formula:

DPPH-scavenging activity (%) = ((A_C_ − A_S_)/A_C_) × 100%

where A_C_ is the absorbance of the control and A_S_ is the absorbance of the sample.

The results were presented as % inhibition of the DPPH radical and Trolox equivalent, calculated from the standard curve. Activity assays were performed in 8 repetitions.

## 5. Conclusions

In the search for pharmacologically active compounds, especially those that are cytotoxic against cancer cell lines and act as antioxidants, we decided to carry out the dimerisation reaction of oleanolic acid (**1**), one of the most popular triterpenes from the oleanane group, exhibiting both cytotoxic activity against cancer cell lines and being an antioxidant. There are dozens of derivatives of the triterpene mentioned above that are known in the scientific literature and exhibit cytotoxic and antioxidant activity. However, obtaining these compounds generally requires multi-stage synthesis and time-consuming, labour-intensive, and cost-intensive purification. The method we have developed for the synthesis of Oleanolic Acid Dimers (OADs) is fast, uncomplicated, does not require expensive, complicated, dangerous, or hard-to-access reagents, and does not require long-term purification, e.g., on a silica gel column, so it is economical in every respect.

Using the method we developed, we obtained 14 OADs (compounds **2a**–**2n**, Figure 1) for which the structures were proven based on spectral data (IR, NMR; Table 6, Table 7 and Table 8). The spectra also confirmed the high degree of purity of OADs obtained after the reaction, with no need for purification, e.g., on a silica gel column, but only by crystallisation. The polarity of the obtained OADs was comparable to that of the parent compound (Table 5).

The structure–activity analysis, carried out using computational methods, showed that the obtained dimers **2a**–**2n** can be effective agents with anticancer and antioxidant properties, demonstrating various mechanisms of this action (Table 1 and Table 2). These results were confirmed by tests on cancer cells (MTT assay) and using the DPPH assay. In the first of the above-mentioned studies, a low IC_50_ value, not exceeding 10 µM, was obtained for most OADs, and for the most active dimer, the IC_50_ value was slightly over 1 µM (Table 3). Additionally, these compounds were characterised by a favourable Selectivity Index value, in many cases of approximately 2 (Table 4). In turn, strong antioxidant properties, especially for OADs with the shortest linkers (Figure 3), were demonstrated for almost all OADs (except for the dimer **2e**). As written earlier, inflammation, especially long-term inflammation, accompanies many diseases and may even cause them, including cancer; therefore, obtaining new, effective agents with potent, cytotoxic, and antioxidative effects at the same time is a precious achievement in the field of medicinal chemistry. An additional advantage is the development of a highly economical method of their synthesis, which allows for obtaining OADs even in large quantities. The OADs we obtained, especially those with the shortest bridges, can be considered as potential anticancer compounds and may become drug candidates.

To summarise, of the 14 OADs obtained (**2a**–**2n**), as many as 13 dimers showed a very high level of cytotoxic activity, with an IC_50_ value below 10 μM. High levels of cytotoxic activity occurred in all four tested cancer cell lines. The most active were dimers containing one, four, seven, and eleven carbon atoms in the linker (OADs **2a**, **2d**, **2e**, **2i**, and **2m**, respectively). In the second stage of assessing the cytotoxic activity of the obtained OADs **2a**–**2n**, the Selectivity Index (SI) values were determined. Among the tested derivatives **2a**–**2n**, the highest SI value was demonstrated by compounds **2c**, **2d**, **2h**, and **2k**, i.e., with a linker containing three, four, six, or nine carbon atoms. In turn, of the fourteen OADs obtained (**2a**–**2n**), three of them, namely **2a**, **2b** (with a one- or two-carbon bridge), and **2f**, showed the highest level of antioxidant activity, with the dimer with the shortest bridge, **2a**, showing by far the highest activity.

Taking into account the above results, the greatest hopes for obtaining highly active and low-toxicity dimer oleanolic acid derivatives should be associated with the synthesis of OADs containing not too many carbon atoms in the linker, preferably no more than four.

In conclusion, the results presented herein offer valuable insights into the cytotoxic activity against cancer cells and the antioxidant potential of synthesised Oleanolic Acid Dimers (OADs, **2a**–**2n**). This study contributes to the ongoing quest for novel cytotoxic and antioxidant agents with therapeutic utility by elucidating structure–activity relationships and identifying compounds with enhanced bioactivity. Further investigations into the derivatives’ mechanisms of action and in vivo efficacy are essential for realising their full pharmaceutical and nutraceutical potential.

## Figures and Tables

**Table 1:**
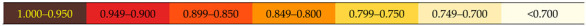
The highest predicted activity of oleanolic acid (**1**) and its dimers **2a**–**2g** determined by the Prediction of Activity Spectra for Substances (PASS) method.

Activity	P_a_ Factor (and P_i_ Factor) of Compounds 1 and 2a–2n							
	OA (1)	2a	2b	2c	2d	2e	2f	2g
**Apoptosis agonist**	0.901 (0.004)	0.905 (0.004)	0.880 (0.005)	0.880 (0.005)	0.867 (0.005)	0.873 (0.005)	0.873 (0.005)	0.865 (0.005)
**Caspase 3 stimulant**	0.984 (0.002)	0.962 (0.003)	0.978 (0.002)	0.978 (0.002)	0.965 (0.002)	0.961 (0.003)	0.961 (0.003)	0.965 (0.002)
**Chemopreventive**	0.937 (0.002)	0.914 (0.002)	0.895 (0.002)	0.895 (0.002)	0.886 (0.003)	0.927 (0.002)	0.927 (0.002)	0.887 (0.003)
**Insulin promoter**	0.869 (0.004)	0.962 (0.002)	0.978 (0.001)	0.978 (0.001)	0.974 (0.001)	0.972 (0.001)	0.972 (0.001)	0.973 (0.001)
**Lipid metabolism** **regulator**	<0.700	<0.700	<0.700	<0.700	0.902 (0.004)	0.906 (0.004)	0.906 (0.004)	0.918 (0.003)
**Lipid peroxidase inhibitor**	0.810 (0.003)	0.852 (0.003)	0.902 (0.004)	0.902 (0.004)	0.902 (0.004)	0.702 (0.005)	0.702 (0.005)	0.782 (0.004)
**Membrane integrity** **antagonist**	0.928 (0.002)	0.884 (0.003)	0.930 (0.002)	0.930 (0.002)	0.934 (0.001)	0.904 (0.003)	0.904 (0.003)	0.937 (0.001)
**Oxidoreductase inhibitor**	0.904 (0.002)	0.866 (0.003)	0.897 (0.002)	0.897 (0.002)	0.897 (0.002)	0.906 (0.002)	0.906 (0.002)	0.900 (0.002)
**Transcription factor NF kappa B stimulant**	0.954 (0.001)	0.930 (0.001)	0.935 (0.001)	0.935 (0.001)	0.930 (0.001)	0.930 (0.001)	0.930 (0.001)	0.927 (0.001)
**Transcription factor** **stimulant**	0.954 (0.001)	0.934 (0.001)	0.935 (0.001)	0.935 (0.001)	0.930 (0.001)	0.930 (0.001)	0.930 (0.001)	0.927 (0.001)

**Legend:** P_a_—probability of activity; P_i_—probability of inactivity.

**Table 2:** The highest predicted activity of oleanolic acid (**1**) and its dimers **2h**–**2n** determined by the Prediction of Activity Spectra for Substances (PASS) method.

Activity	P_a_ Factor (and P_i_ Factor) of Compounds 1 and 2a–2n						
	2h	2i	2j	2k	2l	2m	2n
**Apoptosis agonist**	0.865 (0.005)	0.865 (0.005)	0.865 (0.005)	0.865 (0.005)	0.865 (0.005)	0.865 (0.005)	0.865 (0.005)
**Caspase 3 stimulant**	0.965 (0.002)	0.965 (0.002)	0.965 (0.002)	0.965 (0.002)	0.965 (0.002)	0.965 (0.002)	0.965 (0.002)
**Chemopreventive**	0.887 (0.003)	0.887 (0.003)	0.887 (0.003)	0.887 (0.003)	0.887 (0.003)	0.887 (0.003)	0.887 (0.003)
**Insulin promotor**	0.973 (0.001)	0.973 (0.001)	0.973 (0.001)	0.973 (0.001)	0.973 (0.001)	0.973 (0.001)	0.973 (0.001)
**Lipid metabolism regulator**	0.918 (0.003)	0.918 (0.003)	0.918 (0.003)	0.918 (0.003)	0.918 (0.003)	0.918 (0.003)	0.918 (0.003)
**Lipid peroxidase inhibitor**	0.782 (0.004)	0.782 (0.004)	0.782 (0.004)	0.782 (0.004)	0.782 (0.004)	0.782 (0.004)	0.782 (0.004)
**Membrane integrity antagonist**	0.937 (0.001)	0.937 (0.001)	0.937 (0.001)	0.937 (0.001)	0.937 (0.001)	0.937 (0.001)	0.937 (0.001)
**Oxidoreductase inhibitor**	0.900 (0.002)	0.900 (0.002)	0.900 (0.002)	0.900 (0.002)	0.900 (0.002)	0.900 (0.002)	0.900 (0.002)
**Transcription factor NF kappa B stimulant**	0.927 (0.001)	0.927 (0.001)	0.927 (0.001)	0.927 (0.001)	0.927 (0.001)	0.927 (0.001)	0.927 (0.001)
**Transcription factor** **stimulant**	0.927 (0.001)	0.927 (0.001)	0.927 (0.001)	0.927 (0.001)	0.927 (0.001)	0.927 (0.001)	0.927 (0.001)

**Legend**: **P_a_**—probability of activity; **P_i_**—probability of inactivity.

**Table 3:**
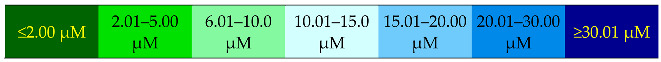
Cytotoxic activity of oleanolic acid (reference compound, **1**) and its dimers (**2a**–**2n**) against the tested cell lines (SKBR-3, SKOV-3, PC-3, U-87, and HDF) determined in the MTT assay and expressed as the half-maximal inhibitory concentration (IC_50_) given in μM (the concentration of a drug or inhibitor needed to inhibit a biological process or response by 50%).

Number of C Atoms in a Linker	Comp. No.	IC_50_ (SD), μM				
		SKBR-3	SKOV-3	PC-3	U-87	HDF
**0**	**1 (OA)**	19.62 (0.02)	18.81 (0.09)	18.63 (0.05)	18.15 (0.01)	24.87 (0.04)
**1**	**2a**	3.96 (0.09)	3.89 (0.01)	3.38 (0.02)	3.32 (0.08)	6.56 (0.08)
**2 sat.**	**2b**	6.67 (0.11)	6.49 (0.01)	6.43 (0.03)	6.59 (0.01)	11.20 (0.03)
**3 sat.**	**2c**	7.64 (0.19)	8.22 (0.01)	7.40 (0.09)	7.46 (0.02)	18.94 (0.06)
**4 sat.**	**2d**	1.12 (0.03)	1.56 (0.01)	1.64 (0.01)	1.20 (0.11)	2.95 (0.01)
**4 unsat. *cis***	**2e**	2.61 (0.01)	2.09 (0.03)	2.27 (0.01)	2.74 (0.09)	2.68 (0.01)
**4 unsat. *trans***	**2f**	5.09 (0.06)	4.79 (0.08)	5.11 (0.01)	4.91 (0.05)	2.78 (0.04)
**5 sat.**	**2g**	5.21 (0.15)	5.16 (0.11)	5.80 (0.04)	5.32 (0.06)	3.37 (0.03)
**6 sat.**	**2h**	6.02 (0.05)	5.39 (0.02)	5.34 (0.07)	5.87 (0.09)	17.31 (0.08)
**7 sat.**	**2i**	3.46 (0.09)	3.68 (0.04)	3.16 (0.08)	3.06 (0.03)	5.39 (0.07)
**8 sat.**	**2j**	9.99 (0.04)	10.27 (0.05)	9.81 (0.02)	10.68 (0.04)	15.96 (0.09)
**9 sat.**	**2k**	6.93 (0.03)	6.89 (0.01)	6.93 (0.05)	7.34 (0.09)	16.82 (0.09)
**10 sat.**	**2l**	18.71 (0.11)	18.37 (0.08)	18.37 (0.07)	18.71 (0.03)	36.27 (0.07)
**11 sat.**	**2m**	3.93 (0.09)	4.58 (0.01)	3.91 (0.06)	4.25 (0.04)	7.43 (0.16)
**12 sat.**	**2n**	6.79 (0.25)	7.41 (0.12)	6.96 (0.04)	6.51 (0.01)	7.30 (0.61)

**Legend**: **OA**—oleanolic acid (reference compound); **sat.**—saturated linker; **unsat.**—unsaturated linker; **IC_50_**—half-maximal inhibitory concentration; **SD**—standard deviation; **SKBR-3**—human breast adenocarcinoma; **SKOV-3**—human ovarian cystadenocarcinoma; **PC-3**—human prostate carcinoma; **U-87**—human glioblastoma; **HDF**—regular fibroblast cell line.

**Table 4:**
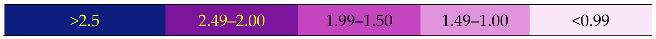
Selectivity Index (SI) of oleanolic acid (reference compound, **1**) and its dimers (**2a**–**2n**) against the tested cell lines (SKBR-3, SKOV-3, PC-3, and U-87) determined in the MTT assay.

Number of C Atoms in a Linker	Comp. No.	SI			
		SKBR-3	SKOV-3	PC-3	U-87
**0**	**1 (OA)**	1.27	1.32	1.33	1.37
**1**	**2a**	1.66	1.69	1.94	1.97
**2 sat.**	**2b**	1.68	1.72	1.74	1.70
**3 sat.**	**2c**	2.48	2.30	2.56	2.54
**4 sat.**	**2d**	2.63	1.89	1.80	2.46
**4 unsat. *cis***	**2e**	1.03	1.28	1.18	0.98
**4 unsat. *trans***	**2f**	0.54	0.58	0.54	0.57
**5 sat.**	**2g**	0.65	0.65	0.58	0.63
**6 sat.**	**2h**	2.87	3.21	3.24	2.95
**7 sat.**	**2i**	1.57	1.46	1.70	1.76
**8 sat.**	**2j**	1.60	1.55	1.63	1.49
**9 sat.**	**2k**	2.43	2.44	2.43	2.29
**10 sat.**	**2l**	1.94	1.97	1.97	1.94
**11 sat.**	**2m**	1.89	1.62	1.90	1.75
**12 sat.**	**2n**	1.07	0.98	1.05	1.12

**Legend**: **OA**—oleanolic acid (reference compound); **sat.**—saturated linker; **unsat.**—unsaturated linker; **SI** = Selectivity Index = IC_50_ for normal cell line (HDF)/IC_50_ for respective cancerous cell line.

**Table 5:**
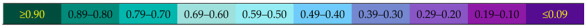
The comparison of R_f_ values for oleanolic acid (**1**) and its dimers (**2a**–**2n**).

Number of C Atoms in a Linker	Comp. No.	R_f_ Value in C_6_H_6_:AcOEt (*v*:*v*)						
		AcOEt	1:1	2:1	4:1	9:1	15:1	25:1
**0**	**1 (OA)**	0.86	0.77	0.62	0.29	0.16	---	---
**1**	**2a**	085	0.74	0.55	0.22	0.13	---	---
**2 sat.**	**2b**	0.85	0.69	0.51	0.18	0.08	---	---
**3 sat**	**2c**	0.85	0.71	0.52	0.18	0.10	---	---
**4 sat.**	**2d**	0.86	0.71	0.52	0.19	0.09	---	---
**4 unsat. *cis***	**2e**	0.86	0.71	0.53	0.19	0.09	---	---
**4 unsat. *trans***	**2f**	0.87	0.73	0.53	0.21	0.10	---	---
**5 sat.**	**2g**	0.87	0.73	0.54	0.22	0.11	---	---
**6 sat.**	**2h**	0.87	0.74	0.55	0.26	0.11	---	---
**7 sat.**	**2i**	0.86	0.76	0.55	0.24	0.10	---	---
**8 sat.**	**2j**	0.91	0.81	0.58	0.21	0.08	---	---
**9 sat.**	**2k**	0.89	0.77	0.60	0.28	0.13	---	---
**10 sat.**	**2l**	0.89	0.78	0.63	0.30	0.15	---	---
**11 sat.**	**2m**	0.90	0.81	0.64	0.32	0.15	---	---
**12 sat.**	**2n**	0.87	0.82	0.64	0.33	0.17	---	---

**Legend**: **OA**—oleanolic acid (reference compound); **sat.**—saturated linker; **unsat.**—unsaturated linker; **R_f_**—retention factor; ***v*:*v***—volume ratio; **AcOEt**—ethyl acetate; **C_6_H_6_**—benzene.

**Table 6:**
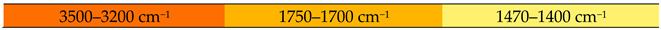
The comparison of wavenumber values (ν) for absorption bands derived from the most characteristic functional groups within molecules of OADs **2a**–**2n**.

Number of C Atoms in a Linker	Comp. No.	Wavenumber Value, ν [cm^−1^]		
		-OH	C=O	C-O-
**0**	**1 (OA)**	3446 *	1687 *	1452 *
**1**	**2a**	3556.01–3205.29	1748.60	1467.60
**2 sat.**	**2b**	3553.84–3203.87	1736.21	1466.29
**3 sat.**	**2c**	3553.84–3203.87	1728.25	1462.19
**4 sat.**	**2d**	3556.00–3201.82	1728.25	1462.19
**4 unsat. *cis***	**2e**	3551.81–3207.99	1726.31	1462.47
**4 unsat. *trans***	**2f**	3550.46–3208.67	1726.32	1462.54
**5 sat.**	**2g**	3554.18–3203.87	1726.32	1462.19
**6 sat.**	**2h**	3557.89–3208.67	1726.32	1462.54
**7 sat.**	**2i**	3557.89–3208.67	1726.21	1463.83
**8 sat.**	**2j**	3557.89–3204.95	1728.25	1462.54
**9 sat.**	**2k**	3553.84–3201.24	1726.16	1462.19
**10 sat.**	**2l**	3554.18–3201.24	1726.32	1462.25
**11 sat.**	**2m**	3554.02–3202.91	1726.34	1462.89
**12 sat.**	**2n**	3553.93–3202.75	1726.77	1463.01

**Legend**: **OA**—oleanolic acid (reference compound); **ν**—wavenumber value; **sat.**—saturated linker; **unsat.**—unsaturated linker; *—published in [36].

**Table 7:**
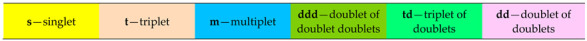
The comparison of chemical shift (δ) values for the most characteristic signals from ^1^H NMR spectra of oleanolic acid (**1**), and OADs **2a**–**2n**.

Number of C Atoms in a Linker	Comp. No.	Chemical Shift, δ [ppm] (Multiplicity, *J* [Hz])			
		C_12_-H	linker	C_3_-H_α_	C_18_-H_β_
**0**	**1 (OA)**	5.27 (t, n.d.) *	---	3.18 (dd, 11.0, 5.0) *	2.85 (dd, 14.0, 4.0) *
**1**	**2a**	5.29 (t, 3.7)	5.74 (s)	3.21 (dd, 10.4, 5.0)	2.83 (dd, 13.7, 4.8)
**2 sat.**	**2b**	5.29 (t, 3.5)	4.30–4.10 (m)	3.21 (dd, 10.3, 4.9)	2.86 (dd, 13.6, 3.7)
**3 sat.**	**2c**	5.28 (t, 3.3)	4.09 (t, 6.2)	3.21 (dd, 11.1, 4.7)	2.85 (dd, 13.7, 3.9)
**4 sat.**	**2d**	5.28 (t, 3.7)	4.30–4.08 (m)	3.21 (dd, 10.4, 4.8)	2.86 (dd, 13.7, 4.8)
**4 unsat. *cis***	**2e**	5.28 (t, 3.5)	4.63 (ddd, 17.8, 13.0, 4.3)	3.21 (dd, 11.1, 4.8)	2.85 (dd, 13.8, 4.0)
**4 unsat. *trans***	**2f**	5.30 (t, 3.4)	4.45 (t, 13.9)	3.16 (dd, 11.5, 3.9)	2.77 (dd, 14.1, 4.1)
**5 sat.**	**2g**	5.29 (t, 3.6)	4.07 (t, 6.5)	3.21 (dd, 11.2, 4.6)	2.86 (dd, 13.6, 4.1)
**6 sat.**	**2h**	5.28 (t, 3.6)	4.01 (t, 6.5)	3.21 (dd, 10.5, 5.0)	2.87 (dd, 13.7, 3.9)
**7 sat.**	**2i**	5.29 (t, 3.5)	4.10–3.94 (m)	3.22 (dd, 11.3, 4.3)	2.87 (dd, 14.0, 4.7)
**8 sat.**	**2j**	5.29 (t, 3.7)	4.02 (td, 6.5, 2.0)	3.25 (dd, 10.8, 4.6)	2.89 (dd, 13.7, 3.8)
**9 sat.**	**2k**	5.29 (t, 3.2)	4.07–3.95 (m)	3.22 (dd, 10.9, 4.6)	2.87 (dd, 13.9, 3.7)
**10 sat.**	**2l**	5.30 (t, 3.4)	4.10–3.93 (m)	3.22 (dd, 11.2, 4.0)	2.88 (dd, 13.8, 4.1)
**11 sat.**	**2m**	5.28 (t, 3.4)	4.00 (td, 6.4, 3.1)	3.21 (dd, 11.0, 4.6)	2.87 (dd, 13.8, 4.1)
**12 sat.**	**2n**	5.28 (t, 3.5)	4.06–3.94 (m)	3.21 (dd, 11.0, 4.6	2.87 (dd, 14.3, 3.7)

**Legend**: **OA**—oleanolic acid (reference compound); **δ**—chemical shift; **sat.**—saturated linker; **unsat.**—unsaturated linker; **n.d.**—no data; ***J***—coupling constant; ***Hz***—hertz (the unit of frequency); *—published in [36].

**Table 8:**
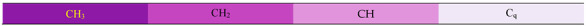
The comparison of chemical shift (δ) values for the most characteristic signals from ^13^C NMR spectra of oleanolic acid (**1**), and OADs **2a**–**2n**.

Number of C Atoms in a Linker	Comp. No.	Chemical Shift, δ [ppm]					
		C-28	C-13	C-12	linker	C-3	C-17
**0**	**OA**	180.4 *	143.79 *	122.25 *	---	78.31 *	45.85 *
**1**	**2a**	176.30	143.29	122.58	79.32	78.95	46.71
**2 sat.**	**2b**	177.44	143.51	122.46	62.15	78.92	46.63
**3 sat.**	**2c**	177.34	143.61	122.20	60.76	78.87	46.65
**4 sat.**	**2d**	177.62	143.74	122.36	63.65	78.91	46.66
**4 unsat. *cis***	**2e**	177.29	143.58	122.38	59.76	78.93	46.61
**4 unsat. *trans***	**2f**	177.33	143.67	122.39	63.72	78.89	46.76
**5 sat.**	**2g**	177.64	143.70	122.29	63.94	78.89	46.59
**6 sat.**	**2h**	177.69	144.01	122.29	64.15	78.82	46.63
**7 sat.**	**2i**	177.64	143.57	122.21	64.05	78.97	46.67
**8 sat.**	**2j**	177.77	143.86	122.34	64.23	79.01	46.68
**9 sat.**	**2k**	177.76	143.82	122.32	64.24	78.96	46.66
**10 sat.**	**2l**	177.77	143.83	122.32	64.25	78.99	46.66
**11 sat.**	**2m**	177.74	143.77	122.24	64.21	78.91	46.62
**12 sat.**	**2n**	177.72	143.75	122.25	64.20	78.89	46.45

**Legend**: **OA**—oleanolic acid (reference compound); **δ**—chemical shift; **sat.**—saturated linker; **unsat.**—unsaturated linker; *—published in [36].

## Data Availability

All data concerning this paper are available in the manuscript body or the Supplementary Data.

## Supplementary Materials

The supporting information can be downloaded at: https://www.mdpi.com/article/10.3390/ijms25136989/s1.
