# Supplementary material for: Oleanolic Acid Dimers with Potential Application in Medicine—Design, Synthesis, Physico-Chemical Characteristics, Cytotoxic and Antioxidant Activity
Source: Int J Mol Sci. 2024 Jun 26;25(13):6989. doi: 10.3390/ijms25136989 (PMC11241395; doi:10.3390/ijms25136989)
Supplement: Supplementary file 1 [file ijms-25-06989-s001.zip › Gunther A. Supplementary Materials/Gunther A. File S1. SAR Analysis of OADs. Improved version.pdf]

Article

Oleanolic Acid Dimers with Potential Application in Medicine – Design, Synthesis, Physico-chemical Characteristics, Cytotoxic and Antioxidant Activity

Andrzej Günther [1,*], Przemysław Zalewski [2,3], Szymon Sip [2], Piotr Ruszkowski [4] and Barbara Bednarczyk-Cwynar [1,5]

[1] Department of Organic Chemistry, Faculty of Pharmacy, Poznan University of Medical Sciences, Collegium Pharmaceuticum 2 (CP.2), Rokietnicka Str. 3, 60–806 Poznan, Poland; e-mail: chem.gunther@gmail.com
[2] Department of Pharmacognosy and Biomaterials, Faculty of Pharmacy, Poznan University of Medical Sciences, Collegium Pharmaceuticum 1 (CP.1), Rokietnicka Str. 3, 60–806 Poznan, Poland; e-mail: pzalewski@ump.edu.pl; szymonsip@ump.edu.pl
[3] Department of Pharmacology and Phytochemistry, Institute of Natural Fibres and Medicinal Plants, 60-630 Poznan, Poland
[4] Department of Pharmacology, Faculty of Pharmacy, Poznan University of Medical Sciences, Collegium Pharmaceuticum 1 (CP.1), Rokietnicka Str. 3, 60–806 Poznan, Poland; e-mail: pruszkowski@gmail.com
[5] Center of Innovative Pharmaceutical Technology (CITF), Rokietnicka Str. 3, 60–806 Poznan, Poland; e-mail: bcwynar@ump.edu.pl
* Correspondence: Andrzej Günther, e-mail: chem.gunther@gmail.com

Supplementary Materials. File S1

Table 1. Predicted activity of oleanolic acid (**1**) and its dimers **2a** – **2n** determined by the PASS method.

1.000 – 0.950	0.949 – 0.900	0.899 – 0.850	0.849 – 0.800	0.799 – 0.750	0.749 – 0.700	< 0.700

	activity	P_a factor (and $P_{i)}$ factor) of oleanolic acid (1) and OADs 2a – 2n for $P_a < 0.700$														
		OA (1)	2a	2b	2c	2d	2e	2f	2g	2h	2i	2j	2k	2l	2m	2n
1	**Acylcarnitine hydrolase inhibitor**	*0.711 (0.025)*	< 0.700	< 0.700	< 0.700	< 0.700	< 0.700	< 0.700	< 0.700	< 0.700	< 0.700	< 0.700	< 0.700	< 0.700	< 0.700	< 0.700
2	**AGPCh hydrolase inhibitor**	*0.825 (0.014)*	< 0.700	0.750 (0.025)	0.750 (0.025)	0.796 (0.018)	< 0.700	< 0.700	0.815 (0.016)	0.815 (0.016)	0.815 (0.016)	0.815 (0.016)	0.815 (0.016)	0.815 (0.016)	0.815 (0.016)	0.815 (0.016)
3	**Antiinflammatory**	*0.819 (0.005)*	0.864 (0.005)	0.814 (0.006)	0.814 (0.006)	0.806 (0.006)	0.775 (0.008)	0.775 (0.008)	0.804 (0.006)	0.804 (0.006)	0.804 (0.006)	0.804 (0.006)	0.804 (0.006)	0.804 (0.006)	0.804 (0.006)	0.804 (0.006)
4	**Antineoplastic**	*0.876 (0.005)*	0.864 (0.006)	0.855 (0.006)	0.855 (0.006)	0.832 (0.008)	0.850 (0.007)	0.850 (0.007)	0.827 (0.009)	0.827 (0.009)	0.827 (0.009)	0.827 (0.009)	0.827 (0.009)	0.827 (0.009)	0.827 (0.009)	0.827 (0.009)

5	**Antineoplastic (lung cancer)**	0.766 (0.005)	0.757 (0.005)	0.746 (0.005)	0.746 (0.005)	0.730 (0.005)	0.728 (0.005)	0.728 (0.005)	0.722 (0.005)	0.722 (0.005)	0.722 (0.005)	0.722 (0.005)	0.722 (0.005)	0.722 (0.005)	0.722 (0.005)	0.722 (0.005)
6	**Antinociceptive**	< 0.700	0.811 (0.002)	0.827 (0.001)	0.827 (0.001)	0.810 (0.002)	0.815 (0.002)	0.815 (0.002)	0.812 (0.002)	0.812 (0.002)	0.812 (0.002)	0.812 (0.002)	0.812 (0.002)	0.812 (0.002)	0.812 (0.002)	0.812 (0.002)
7	**Antiprotozoal (Leishmania)**	0.721 (0.008)	0.724 (0.008)	0.777 (0.005)	0.777 (0.005)	0.806 (0.005)	0.769 (0.006)	0.769 (0.006)	0.821 (0.004)	0.821 (0.004)	0.821 (0.004)	0.821 (0.004)	0.821 (0.004)	0.821 (0.004)	0.821 (0.004)	0.821 (0.004)
8	**Antipruritic**	0.776 (0.004)	< 0.700	0.722 (0.007)	0.722 (0.007)	0.766 (0.004)	0.702 (0.008)	0.702 (0.008)	0.773 (0.004)	0.773 (0.004)	0.773 (0.004)	0.773 (0.004)	0.773 (0.004)	0.773 (0.004)	0.773 (0.004)	0.773 (0.004)
9	**Antisecretoric**	0.707 (0.009)	< 0.700	0.793 (0.004)	0.793 (0.004)	0.801 (0.004)	0.798 (0.004)	0.798 (0.004)	0.818 (0.004)	0.818 (0.004)	0.818 (0.004)	0.818 (0.004)	0.818 (0.004)	0.818 (0.004)	0.818 (0.004)	0.818 (0.004)
10	**Antitussive**	< 0.700	0.704 (0.004)	< 0.700	0.809 (0.003)	< 0.700	< 0.700	< 0.700	< 0.700	< 0.700	< 0.700	< 0.700	< 0.700	< 0.700	< 0.700	< 0.700
11	**Antiviral (Influenza)**	0.836 (0.002)	0.746 (0.004)	0.809 (0.003)	0.809 (0.003)	0.810 (0.003)	0.792 (0.003)	0.792 (0.003)	0.817 (0.003)	0.817 (0.003)	0.817 (0.003)	0.817 (0.003)	0.817 (0.003)	0.817 (0.003)	0.817 (0.003)	0.817 (0.003)
12	**Antiulcerative**	0.771 (0.004)	< 0.700	0.803 (0.004)	0.803 (0.004)	0.800 (0.004)	0.854 (0.003)	0.854 (0.003)	0.811 (0.004)	0.811 (0.004)	0.811 (0.004)	0.811 (0.004)	0.811 (0.004)	0.811 (0.004)	0.811 (0.004)	0.811 (0.004)
13	**Apoptosis agonist**	0.901 (0.004)	0.905 (0.004)	0.880 (0.005)	0.880 (0.005)	0.867 (0.005)	0.873 (0.005)	0.873 (0.005)	0.865 (0.005)	0.865 (0.005)	0.865 (0.005)	0.865 (0.005)	0.865 (0.005)	0.865 (0.005)	0.865 (0.005)	0.865 (0.005)
**14**	**Caspase 3 stimulant**	0.984 (0.002)	0.962 (0.003)	0.978 (0.002)	0.978 (0.002)	0.965 (0.002)	0.961 (0.003)	0.961 (0.003)	0.965 (0.002)	0.965 (0.002)	0.965 (0.002)	0.965 (0.002)	0.965 (0.002)	0.965 (0.002)	0.965 (0.002)	0.965 (0.002)
**15**	**Caspase 8 stimulant**	0.914 (0.001)	0.868 (0.001)	0.896 (0.001)	0.896 (0.001)	0.881 (0.001)	0.859 (0.001)	0.859 (0.001)	0.886 (0.001)	0.886 (0.001)	0.886 (0.001)	0.886 (0.001)	0.886 (0.001)	0.886 (0.001)	0.886 (0.001)	0.886 (0.001)
**16**	**Chemopreventive**	0.937 (0.002)	0.914 (0.002)	0.895 (0.002)	0.895 (0.002)	0.886 (0.003)	0.927 (0.002)	0.927 (0.002)	0.887 (0.003)	0.887 (0.003)	0.887 (0.003)	0.887 (0.003)	0.887 (0.003)	0.887 (0.003)	0.887 (0.003)	0.887 (0.003)
17	**Chitinase inhibitor**	0.789 (0.003)	< 0.700	0.708 (0.005)	0.708 (0.005)	0.750 (0.003)	0.726 (0.004)	0.726 (0.004)	0.756 (0.003)	0.756 (0.003)	0.756 (0.003)	0.756 (0.003)	0.756 (0.003)	0.756 (0.003)	0.756 (0.003)	0.756 (0.003)
18	**CYP2J substrate**	0.808 (0.019)	0.712 (0.046)	0.712 (0.046)	0.712 (0.046)	< 0.700	0.708 (0.048)	0.708 (0.048)	0.712 (0.046)	0.712 (0.046)	0.712 (0.046)	0.712 (0.046)	0.712 (0.046)	0.712 (0.046)	0.712 (0.046)	0.712 (0.046)
19	**Cytoprotectant**	0.709 (0.004)	< 0.700	0.708 (0.005)	0.708 (0.005)	0.715 (0.004)	0.763 (0.003)	0.763 (0.003)	0.722 (0.004)	0.722 (0.004)	0.722 (0.004)	0.722 (0.004)	0.722 (0.004)	< 0.700	0.722 (0.004)	0.722 (0.004)
20	**DAGOA transferase inhibitor**	< 0.700	< 0.700	0.792 (0.001)	0.792 (0.001)	0.822 (0.001)	< 0.700	< 0.700	0.834 (0.001)	0.834 (0.001)	0.834 (0.001)	0.834 (0.001)	0.834 (0.001)	0.834 (0.001)	0.834 (0.001)	0.834 (0.001)
21	**DNA ligase (ATP) inhibitor**	0.803 (0.001)	0.723 (0.001)	0.756 (0.001)	0.756 (0.001)	0.731 (0.001)	0.735 (0.001)	0.735 (0.001)	0.733 (0.001)	0.733 (0.001)	0.733 (0.001)	0.733 (0.001)	0.733 (0.001)	0.733 (0.001)	0.733 (0.001)	0.733 (0.001)
22	**Gastrin inhibitor**	< 0.700	< 0.700	< 0.700	< 0.700	0.703 (0.005)	< 0.700	< 0.700	0.712 (0.004)	0.712 (0.004)	0.712 (0.004)	0.712 (0.004)	0.712 (0.004)	< 0.700	0.712 (0.004)	0.712 (0.004)
23	**Hepatic disorders**	0.834	0.813	0.823	0.823	0.820	0.825	0.825	< 0.700	0.815	0.815	0.815	0.815	0.815	0.815	0.815

	**treatment**	*(0.004)*	(0.004)	(0.004)	(0.004)	(0.004)	(0.004)	(0.004)		(0.004)	(0.004)	(0.004)	(0.004)	(0.004)	(0.004)	(0.004)
**24**	**Hepatoprotectant**	*0.930 (0.002)*	0.917 (0.002)	0.942 (0.002)	0.942 (0.002)	0.961 (0.001)	0.961 (0.001)	0.961 (0.001)	0.964 (0.001)	0.964 (0.001)	0.964 (0.001)	0.964 (0.001)	0.964 (0.001)	0.964 (0.001)	0.964 (0.001)	0.964 (0.001)
25	**Hypolipemic**	*0.713 (0.013)*	0.778 (0.008)	0.829 (0.006)	0.829 (0.006)	0.851 (0.005)	0.866 (0.005)	< 0.700	0.865 (0.005)	0.865 (0.005)	0.865 (0.005)	0.865 (0.005)	0.865 (0.005)	0.865 (0.005)	0.865 (0.005)	0.865 (0.005)
26	**ICAM1 expression inhibitor**	*0.747 (0.001)*	< 0.700	0.715 (0.002)	0.715 (0.002)	0.709 (0.002)	< 0.700	< 0.700	< 0.700	< 0.700	< 0.700	< 0.700	< 0.700	< 0.700	< 0.700	< 0.700
**27**	**Insulin promotor**	*0.869 (0.004)*	0.962 (0.002)	0.978 (0.001)	0.978 (0.001)	0.974 (0.001)	0.972 (0.001)	0.972 (0.001)	0.973 (0.001)	0.973 (0.001)	0.973 (0.001)	0.973 (0.001)	0.973 (0.001)	0.973 (0.001)	0.973 (0.001)	0.973 (0.001)
**28**	**Lipid metabolism regulator**	*< 0.700*	< 0.700	< 0.700	< 0.700	0.902 (0.004)	0.906 (0.004)	0.906 (0.004)	0.918 (0.003)	0.918 (0.003)	0.918 (0.003)	0.918 (0.003)	0.918 (0.003)	0.918 (0.003)	0.918 (0.003)	0.918 (0.003)
**29**	**Lipid peroxidase inhibitor**	*0.810 (0.003)*	0.852 (0.003)	0.902 (0.004)	0.902 (0.004)	0.902 (0.004)	0.702 (0.005)	0.702 (0.005)	0.782 (0.004)	0.782 (0.004)	0.782 (0.004)	0.782 (0.004)	0.782 (0.004)	0.782 (0.004)	0.782 (0.004)	0.782 (0.004)
**30**	**Membrane integrity antagonist**	*0.928 (0.002)*	0.884 (0.003)	0.930 (0.002)	0.930 (0.002)	0.934 (0.001)	0.904 (0.003)	0.904 (0.003)	0.937 (0.001)	0.937 (0.001)	0.937 (0.001)	0.937 (0.001)	0.937 (0.001)	0.937 (0.001)	0.937 (0.001)	0.937 (0.001)
31	**Mucomembranous protectant**	*0.894 (0.005)*	0.778 (0.025)	0.825 (0.013)	0.825 (0.013)	0.803 (0.018)	0.815 (0.015)	0.815 (0.015)	0.810 (0.016)	0.810 (0.016)	0.810 (0.016)	0.810 (0.016)	0.810 (0.016)	0.810 (0.016)	0.810 (0.016)	0.810 (0.016)
32	**Nitric oxide antagonist**	*0.814 (0.002)*	0.796 (0.003)	0.784 (0.003)	0.784 (0.003)	0.766 (0.003)	0.769 (0.003)	0.769 (0.003)	0.758 (0.003)	0.758 (0.003)	0.758 (0.003)	0.758 (0.003)	0.758 (0.003)	0.758 (0.003)	0.758 (0.003)	0.758 (0.003)
**33**	**Oxidoreductase inhibitor**	*0.904 (0.002)*	0.866 (0.003)	0.897 (0.002)	0.897 (0.002)	0.897 (0.002)	0.906 (0.002)	0.906 (0.002)	0.900 (0.002)	0.900 (0.002)	0.900 (0.002)	0.900 (0.002)	0.900 (0.002)	0.900 (0.002)	0.900 (0.002)	0.900 (0.002)
34	**Phospholipase C inhibitor**	*< 0.700*	< 0.700	< 0.700	< 0.700	0.798 (0.002)	< 0.700	< 0.700	0.844 (0.002)	0.844 (0.002)	0.844 (0.002)	0.844 (0.002)	0.844 (0.002)	0.844 (0.002)	0.844 (0.002)	0.844 (0.002)
35	**Phosphatase inhibitor**	*0.894 (0.001)*	0.730 (0.008)	0.739 (0.007)	0.739 (0.007)	0.731 (0.008)	0.733 (0.008)	0.733 (0.008)	0.726 (0.009)	0.726 (0.009)	0.726 (0.009)	0.726 (0.009)	0.726 (0.009)	0.726 (0.009)	0.726 (0.009)	0.726 (0.009)
36	**Protein phosphatase inhibitor**	*0.782 (0.002)*	< 0.700	0.713 (0.003)	0.713 (0.003)	< 0.700	0.709 (0.003)	0.709 (0.003)	< 0.700	< 0.700	< 0.700	< 0.700	< 0.700	< 0.700	< 0.700	< 0.700
37	**PT phosphatase inhibitor**	*0.764 (0.002)*	< 0.700	< 0.700	< 0.700	< 0.700	< 0.700	< 0.700	< 0.700	< 0.700	< 0.700	< 0.700	< 0.700	< 0.700	< 0.700	< 0.700
38	**T-cell PT phosphatase inhibitor**	*0.726 (0.001)*	< 0.700	< 0.700	< 0.700	< 0.700	< 0.700	< 0.700	< 0.700	< 0.700	< 0.700	< 0.700	< 0.700	< 0.700	< 0.700	< 0.700
39	**T-17β-dehydr. (NADP+) inhibitor**	*0.892 (0.007)*	0.775 (0.032)	0.775 (0.032	0.775 (0.032)	0.758 (0.037)	0.719 (0.049)	0.719 (0.049)	0.730 (0.045)	0.730 (0.045)	0.730 (0.045)	< 0.700	0.730 (0.045)	0.730 (0.045)	0.730 (0.045)	0.730 (0.045)
**40**	**Transcription factor NF kappa B stimulant**	*0.954 (0.001)*	0.930 (0.001)	0.935 (0.001)	0.935 (0.001)	0.930 (0.001)	0.930 (0.001)	0.930 (0.001)	0.927 (0.001)	0.927 (0.001)	0.927 (0.001)	0.927 (0.001)	0.927 (0.001)	0.927 (0.001)	0.927 (0.001)	0.927 (0.001)
**41**	**Transcription factor stimulant**	*0.954 (0.001)*	0.934 (0.001)	0.935 (0.001)	0.935 (0.001)	0.930 (0.001)	0.930 (0.001)	0.930 (0.001)	0.927 (0.001)	0.927 (0.001)	0.927 (0.001)	0.927 (0.001)	0.927 (0.001)	0.927 (0.001)	0.927 (0.001)	0.927 (0.001)

42	**Vasodilator peripheral**	*< 0.700*	0.717 (0.008)	< 0.700	< 0.700	< 0.700	< 0.700	< 0.700	< 0.700	< 0.700	< 0.700	< 0.700	< 0.700	< 0.700	< 0.700	< 0.700
43	**Wound healing agent**	*< 0.700*	0.735 (0.004)	< 0.700	< 0.700	< 0.700	< 0.700	< 0.700	< 0.700	< 0.700	< 0.700	< 0.700	< 0.700	< 0.700	< 0.700	< 0.700

Legend: **P_a** = probability of activity; **P_i** = probability of inactivity; **AGPCh** = alkenylglycerophosphocholine; **DAGOA**= diacylglycerol O-acyl; **PT** = protein-tyrosine; **T-17β-dehydr.** = testosterone 17beta-dehydrogenase
