# Supplementary figures and images for: Oleanolic Acid Dimers with Potential Application in Medicine—Design, Synthesis, Physico-Chemical Characteristics, Cytotoxic and Antioxidant Activity
Source: Int J Mol Sci. 2024 Jun 26;25(13):6989. doi: 10.3390/ijms25136989 (PMC11241395; doi:10.3390/ijms25136989)

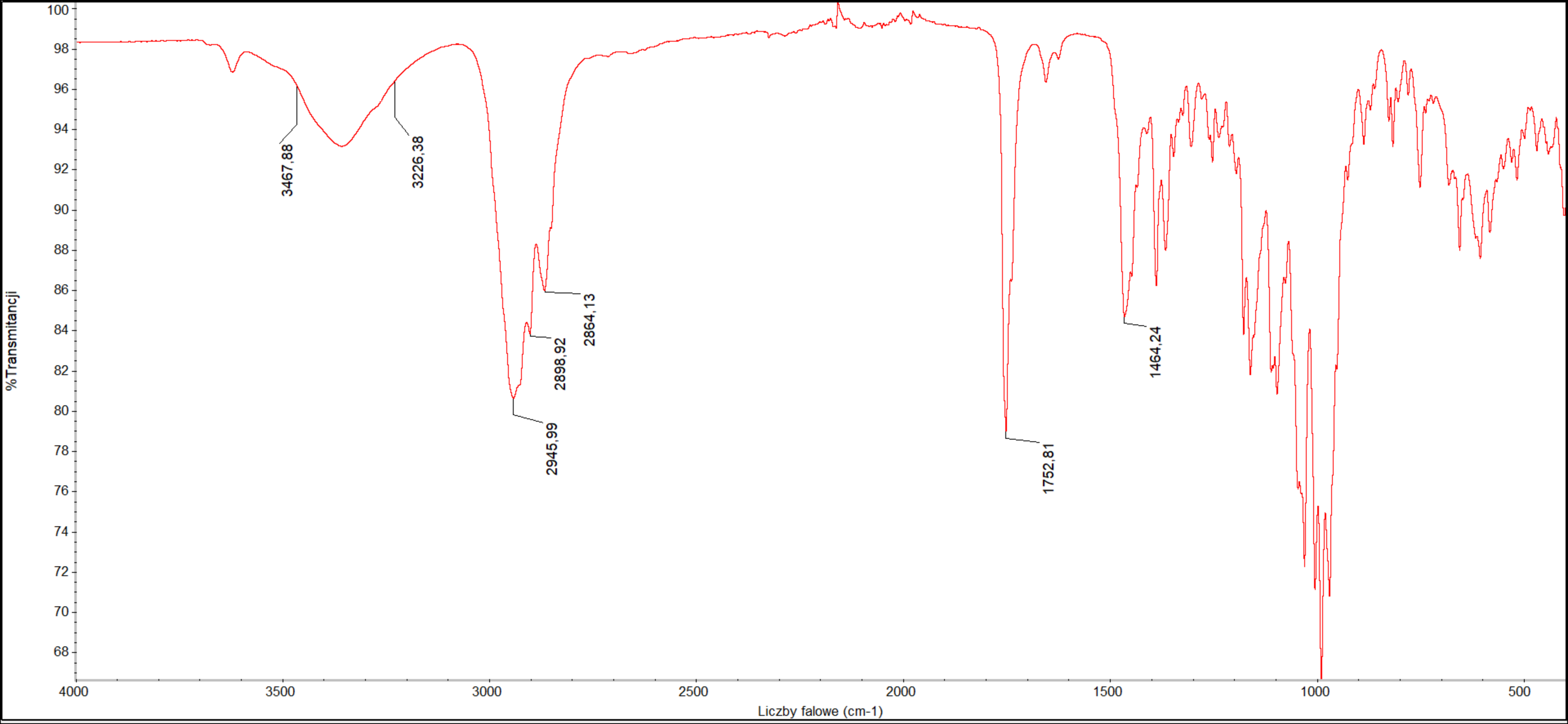

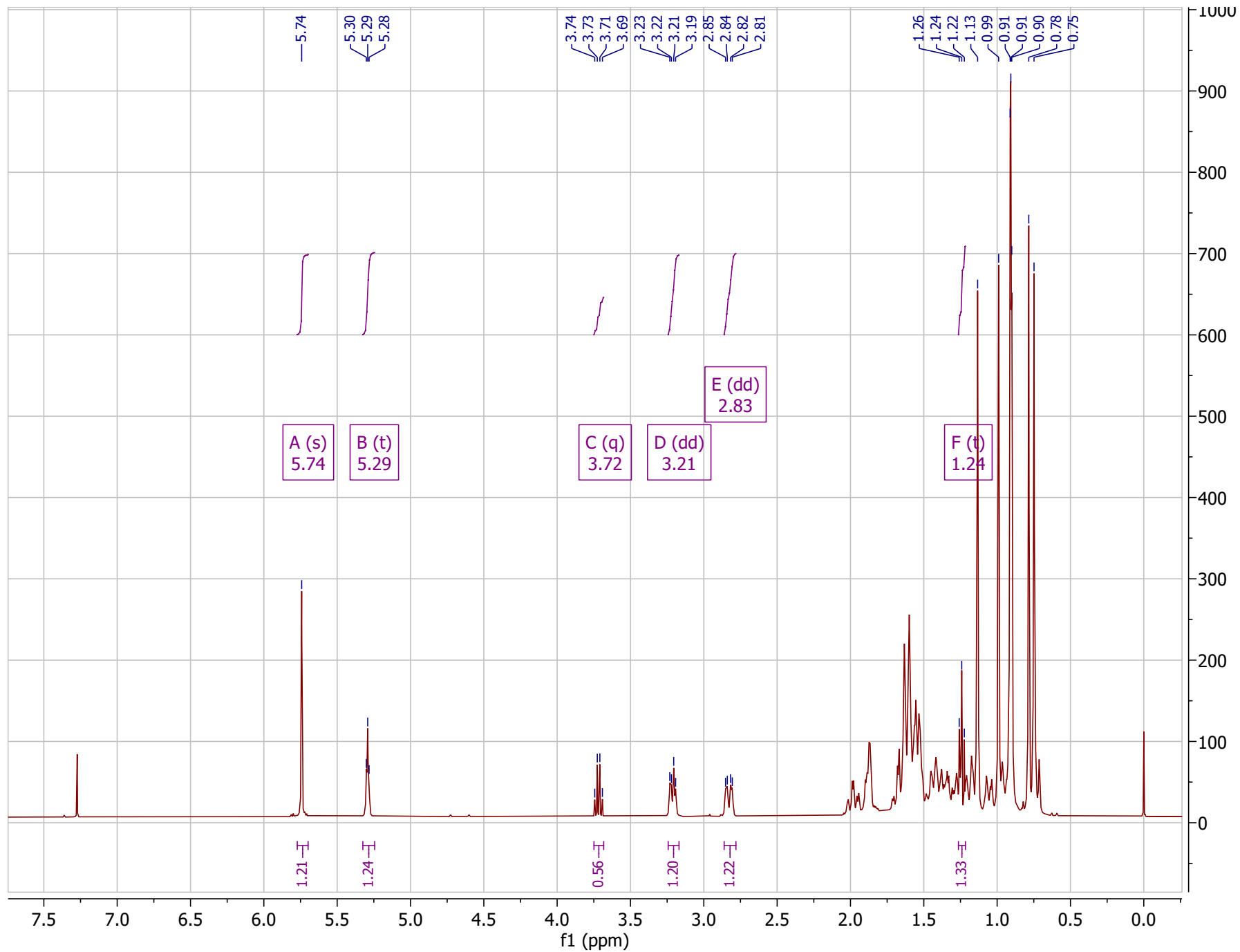

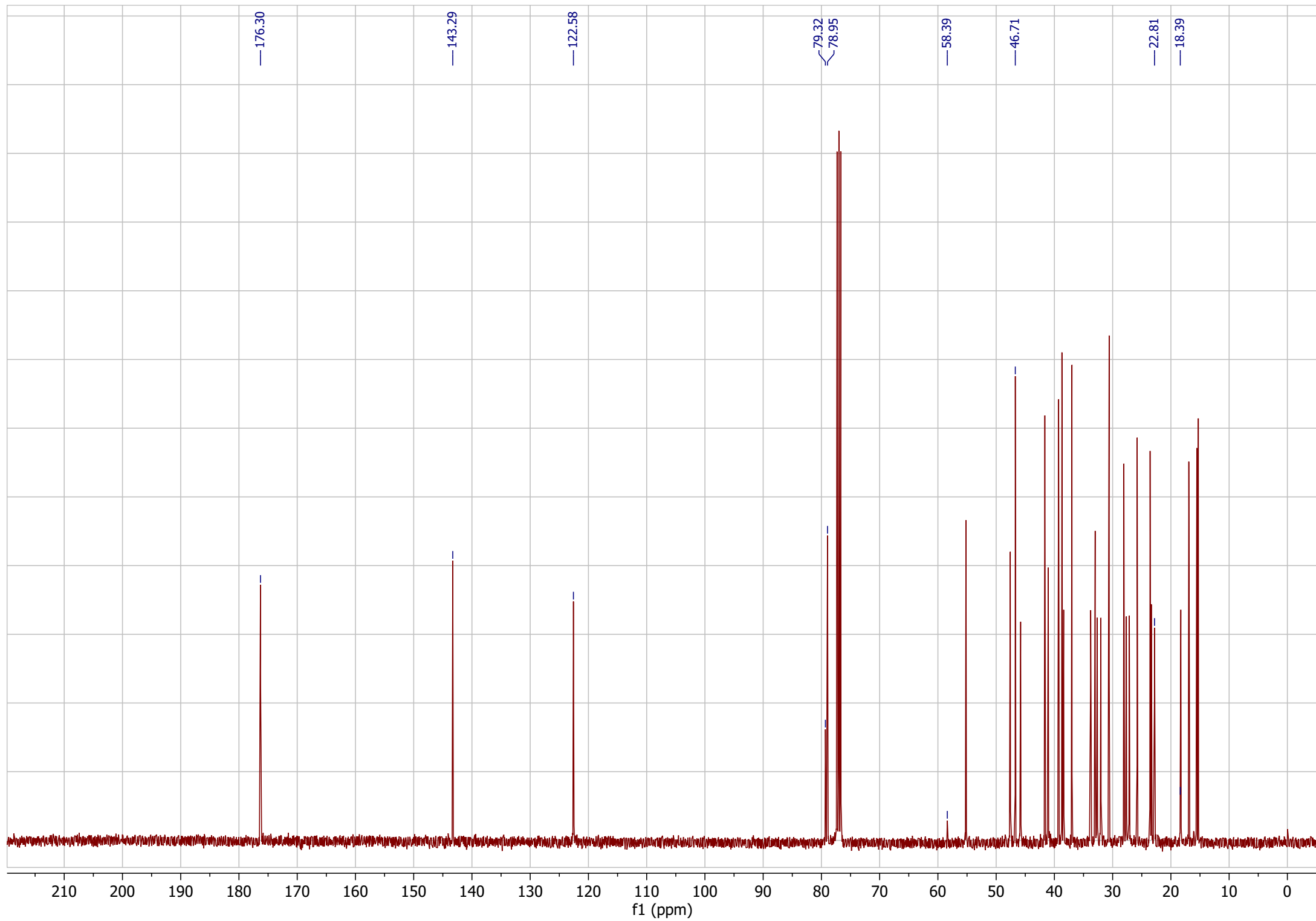

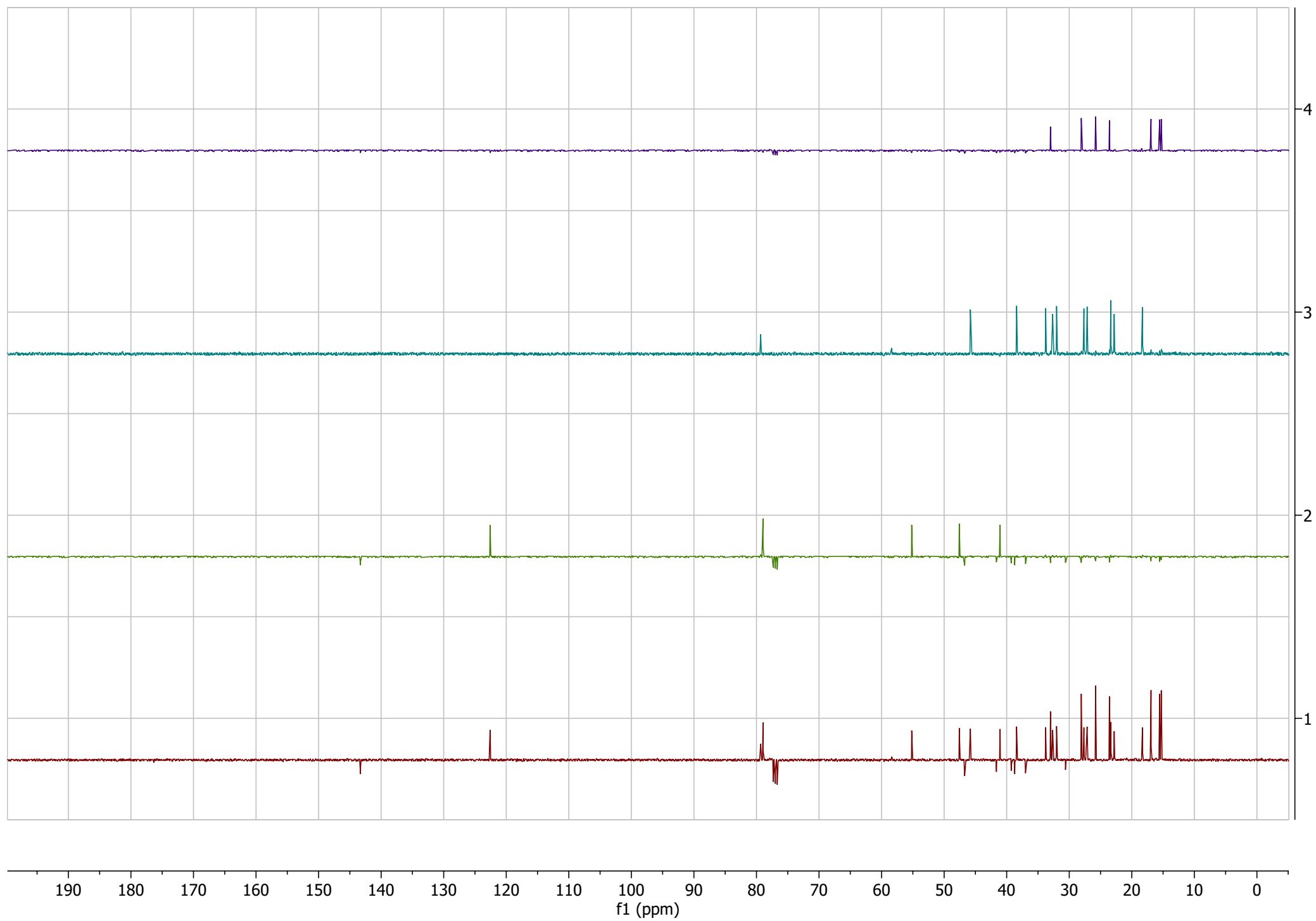

Supplement: Supplementary file 1 [file ijms-25-06989-s001.zip › Gunther A. Supplementary Materials/Gunther A. File S2. Dimer 2a, IR and NMR spectra.pdf]
